# Adipose tissue–derived MFG-E8 promotes hepatic inflammation and fibrosis through macrophage activation in a mouse MASH model

**DOI:** 10.1038/s44324-026-00099-0

**Published:** 2026-02-20

**Authors:** Masashi Kuroda, Kazuhiro Nomura, Azumi Wada, Yui Hatano, Miki Ogawa, Saya Okamoto, Etsuko Ishikawa, Yuna Izumi-Mishima, Sonoko Yasui-Yamada, Yasuo M. Tsutsumi, Nagakatsu Harada, Rie Tsutsumi, Hiroshi Sakaue

**Affiliations:** 1 Department of Nutrition and Metabolism, Institute of Biomedical Sciences, Tokushima University Graduate School, Tokushima City, Tokushima Japan; 2 Diabetes Therapeutics and Research Center, Tokushima University, Tokushima City, Tokushima Japan; 3 Department of Anesthesiology and Critical Care, Hiroshima University, Hiroshima City, Hiroshima Japan; 4 Department of Health and Nutrition, Faculty of Nursing and Nutrition, The University of Shimane, Izumo City, Shimane Japan

**Keywords:** Diseases, Gastroenterology, Immunology

## Abstract

Metabolic dysfunction–associated steatohepatitis (MASH) is characterized by hepatocellular injury, macrophage activation, and severe fibrosis, and often progresses to liver cirrhosis and hepatocellular carcinoma. Excessive accumulation of visceral fat exacerbates hepatic inflammation and fibrosis independently of fatty liver, but the underlying molecular mechanisms have remained unclear. We here identify MFG-E8 (milk fat globule–EGF8) as a secreted protein that is overexpressed in adipose tissue of obese mice and contributes to such exacerbation. MFG-E8 deficiency in MASH model (STAM-MASH) mice was associated with reduced hepatic expression of inflammation- and fibrosis-related genes without attenuation of steatosis. Conversely, MFG-E8 supplementation in MFG-E8 knockout mice intensified hepatic inflammation and promoted the formation of hepatic crownlike structures. Coculture of macrophages with apoptotic hepatocytes induced expression of inflammatory cytokine genes, and this effect was enhanced by the presence of exogenous MFG-E8 in the culture medium. Our findings suggest that adipose tissue–derived MFG-E8 infiltrates the liver and promotes macrophage-hepatocyte interaction, thereby contributing to hepatic inflammation and fibrosis in MASH.

## Introduction

The worldwide prevalence of metabolic dysfunction–associated steatotic liver disease (MASLD) and metabolic dysfunction–associated steatohepatitis (MASH) is estimated to be 38.0% and 5.27%, respectively^1^. The progression of MASLD to MASH is characterized by the development of hepatitis and liver fibrosis in addition to hepatic steatosis, with liver fibrosis, in particular, increasing the risk of complications such as cirrhosis, liver failure, hepatocellular carcinoma, and extrahepatic conditions including cardiovascular disease, chronic kidney disease, and certain other types of cancer^2,3^.

The widely accepted two-hit hypothesis proposes that MASH results from excessive hepatic fat accumulation (first hit) and chronic inflammation and fibrosis induced by various factors that damage hepatocytes (second hit)^4^. Cytokines released from activated monocyte-derived macrophages—such as tumor necrosis factor (TNF)–α, transforming growth factor (TGF)–β, and platelet-derived growth factor (PDGF)—contribute to the exacerbation of inflammation, the activation of hepatic stellate cells and their differentiation into fibroblasts, and the overproduction of extracellular matrix (ECM).

Cell death has recently attracted attention as one mechanism of such macrophage activation. During MASH progression, hepatocytes undergo cell death as a result of various stresses imposed by fat accumulation, and these dead cells are eliminated by macrophages through phagocytosis. Indeed, hepatic crownlike structures (hCLSs), in which dead hepatocytes are surrounded by macrophages, are frequently observed in MASH models^5,6^. Activated fibroblasts and collagen deposition are also observed around hCLSs, and the number of hCLSs is positively correlated with the area of fibrosis in the liver. It has therefore been proposed that hCLSs are a site of interaction between macrophages and dead cells and that such interaction may directly activate macrophages, thereby giving rise to MASH progression from simple steatosis^5,6^.

On the other hand, visceral adiposity is also a key factor in MASH pathogenesis, being observed in >90% of affected individuals. Fatty acids released from the increased amounts of adipose tissue may enter the liver via the portal vein and promote the development of fatty liver. However, a relation between visceral fat volume and liver inflammation and fibrosis remains apparent even after adjustment for the extent of fatty liver^7^, suggesting the existence of a mechanism by which visceral fat may promote MASH progression independently of hepatic fat accumulation. Factors derived from adipose tissue in obese individuals may therefore regulate immune cells and fibrosis in the liver, but such factors have remained to be identified.

We here performed DNA microarray analysis of mouse adipose tissue to identify adipose-derived humoral factors that might contribute to MASH pathogenesis, and we thereby identified the gene for milk fat globule–epidermal growth factor 8 (MFG-E8) among the genes whose expression was induced by consumption of a high-fat diet (HFD). MFG-E8 is a glycoprotein that was originally identified as a component of the milk fat globule membrane^8^. In rodents, it is expressed as splice variants with a molecular size of up to 66 kDa, with each variant containing two epidermal growth factor (EGF)–like domains, an RGD motif that binds to αvβ3 or αvβ5 integrins on phagocytes, and a discoidin domain that binds to phosphatidylserine (PS) on apoptotic cells. Through such binding to these cells, MFG-E8 promotes the efficient uptake and removal of dead cells by phagocytes^9^. Given this function of MFG-E8 in mediating interaction between macrophages and dead cells, we investigated the possibility that adipose-derived MFG-E8 contributes to the activation of hepatic macrophages and the development of fibrosis in MASH.

## Results

### MFG-E8 expression in adipose tissue of STAM-MASH mice

DNA microarray analysis of epididymal white adipose tissue (eWAT) from 16-week-old mice revealed 420 genes that were upregulated in obese animals that had been fed a HFD from 9 weeks of age compared with control animals maintained on a normal chow (NC) diet (Supplementary Fig. 1a). The DAVID functional annotation tool identified 65 of these 420 genes as encoding secreted proteins. From among these 65 genes, we then excluded those with higher expression levels in liver than in visceral fat on the basis of expression status obtained from the GTEx portal (https://www.gtexportal.org/home), leaving 52 genes. These remaining genes were further narrowed down to 22 genes on the basis that the blood concentrations of their encoded proteins were found to be increased in humans with obesity or related diseases (insulin resistance or type 2 diabetes) in previous studies^10–34^(Supplementary Fig. 1b). In the present study, we determined to focus on MFG-E8 among these 22 proteins on the basis of its functional characteristics.

**Fig. 1:**
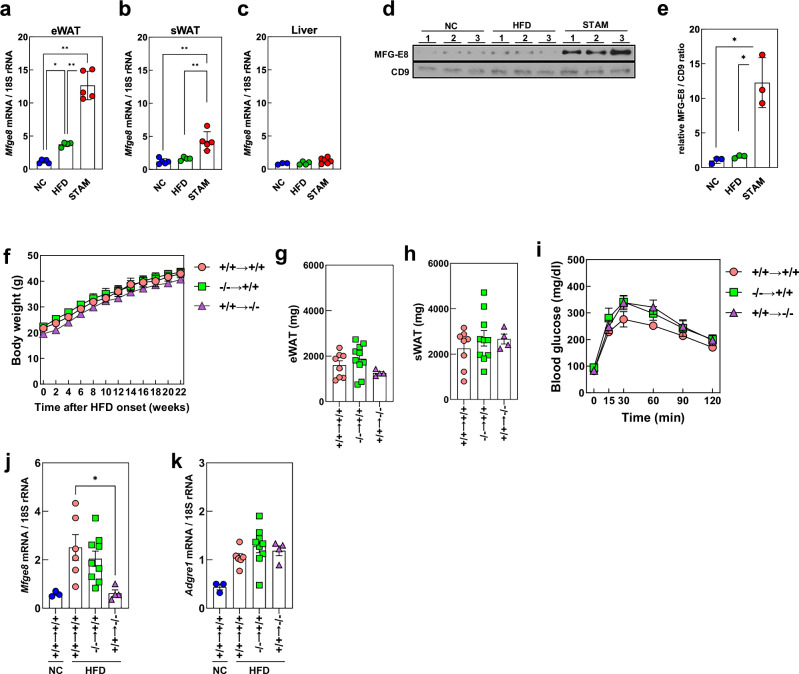
MFG-E8 expression in HFD-fed and STAM-MASH mice. **a** RT–qPCR analysis of *Mfge8* mRNA abundance in epididymal white adipose tissue (eWAT) (*n* = 4 or 5). **b** RT–qPCR analysis of *Mfge8* mRNA abundance in subcutaneous white adipose tissue (sWAT) (*n* = 4 or 5). **c** RT–qPCR analysis of *Mfge8* mRNA abundance in liver (*n* = 4 or 5). **d** Immunoblot analysis of MFG-E8 and CD9 in circulating extracellular vesicles (EVs) isolated from normal chow (NC)-fed, high-fat diet (HFD)-fed, or STAM-MASH mice (*n* = 3). **e** Quantification of the MFG-E8/CD9 ratio relative to NC-fed mice (*n* = 3). **f** Time course of body weight in bone-marrow-transplanted (BMT) mice after initiation of HFD feeding (*n* = 4–8). **g** eWAT weight in BMT recipient mice after 22 weeks of HFD feeding (*n* = 4–8). **h** sWAT weight in BMT recipient mice after 22 weeks of HFD feeding (*n* = 4–8). **i** Oral glucose-tolerance test at 12 weeks after initiation of HFD feeding (*n* = 4–8). Mice were fasted for 18 h before glucose administration (2 g/kg). **j** RT–qPCR analysis of *Mfge8* mRNA abundance in eWAT of BMT recipient mice after 22 weeks of HFD or NC feeding (*n* = 3–8). **k** RT–qPCR analysis of *Adgre1* mRNA abundance in eWAT of BMT recipient mice after 22 weeks of HFD or NC feeding (*n* = 3–8). In (**a**–**e**), marker colors indicate the experimental groups: blue, NC-fed mice; green, HFD-fed mice; red, STAM-MASH mice. In (**f**–**k**), marker shapes and colors indicate donor–recipient combinations in the BMT experiment (donor → recipient): pink circles, wild type (WT) → WT (+/+→+/+); green squares, Mfge8 knockout (KO) → WT (–/– → +/+); purple triangles, WT → KO (+/+ → −/−). Blue circles indicate NC-fed (+/+→+/+) controls. The amounts of mRNAs were normalized to 18S rRNA. Quantitative data are shown as means ± s.e.m. *P < 0.05; **P < 0.01 (one-way ANOVA with Tukey’s post-hoc test).

We first examined expression of the MFG-E8 gene (*Mfge8*) in various tissues of mice fed a NC or a HFD and of STAM-MASH mice (Fig. 1a–c, Supplementary Fig. 1c). RT–qPCR analysis revealed that expression of *Mfge8* in eWAT was increased in HFD-fed mice compared with NC-fed mice, and that it was increased further in STAM-MASH mice, whereas expression levels in the liver did not differ significantly among the three mouse groups. Expression of *Mfge8* was also prominent in the spleen of HFD-fed mice, but it was not increased further in STAM-MASH mice. We next investigated whether this selective induction of adipose *Mfge8* expression might result in an increase in the blood concentration of the encoded protein. Given that circulating MFG-E8 protein is bound to extracellular vesicles (EVs) via phosphatidylserine on their surface^35^, we isolated EVs from serum by ultracentrifugation and quantified the EV-associated MFG-E8 by immunoblot analysis. The circulating concentration of MFG-E8 was much higher in STAM-MASH mice than in mice fed a NC or a HFD (Fig. 1d, e).

Fractionation of eWAT revealed that both the SVF and adipocytes expressed *Mfge8* in NC-fed mice, but that the expression level was selectively increased in the adipocyte fraction of HFD-fed mice and STAM-MASH mice (Supplementary Fig. 2a). Given that the expression of adipocytokines is dependent on fat accumulation in adipocytes^36^, we examined whether the same might be true for the expression of MFG-E8. Although STAM-MASH mice had lower or comparable body weight and adipose tissue weight compared to NC-fed mice (Supplementary Fig. 2b,c), we confirmed that the diameter of adipocytes was greater for HFD-fed and STAM-MASH mice than for NC-fed mice (Supplementary Fig. 2d,e). To exclude the effects of hormonal changes associated with obesity, we also examined the expression of *Mfge8* in 3T3-L1 cells that had been induced to differentiate into adipocytes and maintained for up to 20 days. We previously showed that both the size of lipid droplets in these cells and overall cell size increased with duration of the culture period^37^. RT–qPCR analysis indeed revealed that adipogenic differentiation of 3T3-L1 cells was accompanied by an increase in *Mfge8* expression, and that this increase tended to be greater in cells maintained for 20 days than in those maintained for 6 days (Supplementary Fig. 2f). To validate these findings, we reanalyzed publicly available single-cell RNA-seq datasets from obese mice^38,39^. In the liver, *Mfge8* expression changed in hepatocyte precursors and myofibroblasts, with increased expression observed in myofibroblasts specifically as the duration of high-calorie dietary prolonged (Supplementary Fig. 2g)^38^. In adipose tissue, both the proportion of *Mfge8*–positive cells and the mean expression level tended to increase in some cell populations (Supplementary Fig. 2h)^39^. Notably, although not statistically significant, adipocytes also exhibited a trend toward increased *Mfge8* expression under HFD conditions. While these datasets were derived from models distinct from ours and should therefore be interpreted as supportive evidence, the analysis suggests that adipocytes represent one of the major cellular sources of *Mfge8* in obesity-associated states.

To further confirm that the major source of MFG-E8 in adipose tissue of obese mice is adipocytes, we performed bone marrow transplantation experiments with wild-type (WT) and MFG-E8 global knockout (KO) mice. Fat accumulation and glucose metabolism did not appear to differ among the various combinations of genotypes for donor and recipient in these experiments (Fig. 1f–i). Furthermore, the HFD-induced expression of *Mfge8* in eWAT was similarly observed in WT mice whose bone marrow cells had been replaced with those from KO or WT mice (Fig. 1j). On the other hand, no such induction of *Mfge8* expression was apparent in KO mouse recipients of WT bone marrow cells. Since *Adgre1* macrophage marker level was comparable, suggesting that host and donor genotype did not affect adipose macrophage number (Fig. 1k). These results thus indicated that adipocytes were responsible for the increase in *Mfge8* expression apparent in adipose tissue of HFD-fed or STAM-MASH mice.

### Amelioration of MASH in MFG-E8 KO mice

**Fig. 2:**
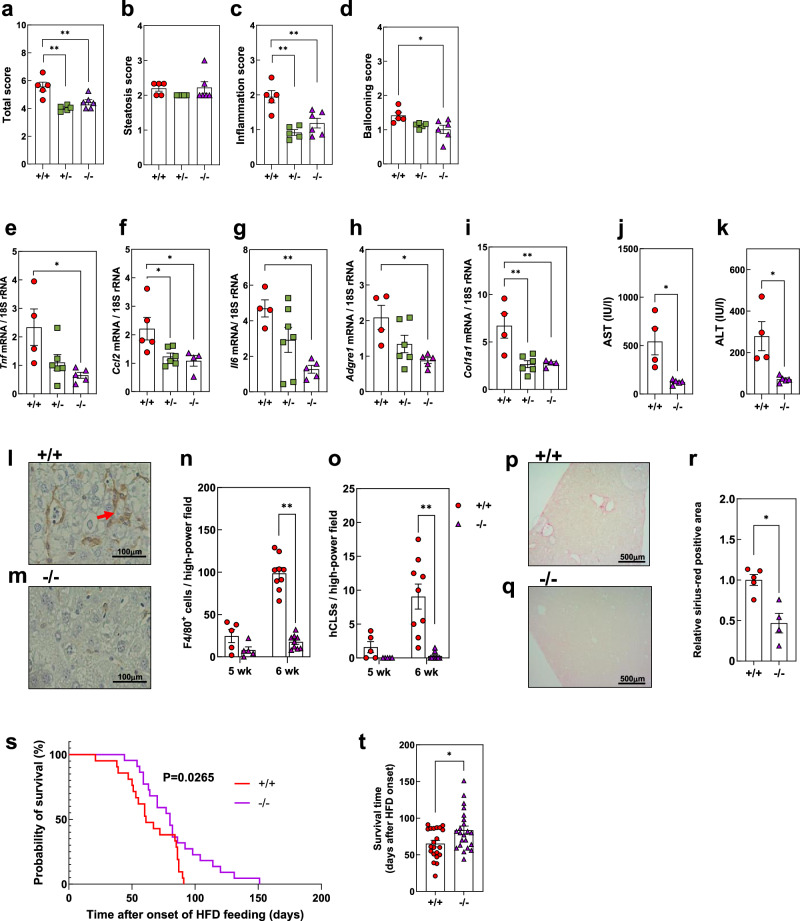
Effects of MFG-E8 deficiency in the STAM-MASH model. **a** Total NAS scores (*n* = 5–6). **b** Steatosis scores (*n* = 5–6). **c** Inflammation scores (*n* = 5–6). **d** Ballooning scores (*n* = 5–6). **e** RT–qPCR analysis of *Tnf* mRNA abundance in liver (*n* = 4–5). **f** RT–qPCR analysis of *Ccl2* mRNA abundance in liver (*n* = 4–5). **g** RT–qPCR analysis of *Il6* mRNA abundance in liver (*n* = 4–5). **h**, RT–qPCR analysis of *Adgre1* mRNA abundance in liver (*n* = 4–5). **i** RT–qPCR analysis of *Col1a1* mRNA abundance in liver (*n* = 4–5). **j** Serum aspartate aminotransferase (AST) levels (*n* = 4–5). **k** Serum alanine aminotransferase (ALT) levels (*n* = 4–5). **l** Representative F4/80 immunohistochemical staining in liver of WT mice (+/+). **m** Representative F4/80 staining in liver of MFG-E8 KO mice (–/–). **n** Quantification of F4/80-positive cells per high-power field (0.2 mm²) (*n* = 5–9). **o** Quantification of hepatic crown-like structures (hCLS) per high-power field (0.2 mm²) (*n* = 5–9). **p** Representative Sirius Red–stained liver section from WT mice. **q** Representative Sirius Red–stained liver section from MFG-E8 KO mice. **r** Quantification of relative Sirius Red–positive area (*n* = 4–5). **s** Kaplan–Meier survival curves in the STAM-MASH model (*n* = 21–22). Line colors in the panel indicate the genotype: red, WT (+/+); purple, MFG-E8 KO (–/–). **t** Survival duration in the STAM-MASH model (*n* = 21–22). Throughout Fig. 2, markers indicate the genotype of STAM-MASH mice: red circles, WT (+/+); green squares, heterozygous MFG-E8 knockout (+/−); purple triangles, homozygous knockout (−/−). Panels (**l**–**o**) show 5–6-week-old mice, which are younger than the 8-week-old animals used in most other experiments. The *P*-value in panel s was determined using the log-rank test. The amounts of mRNAs were normalized to 18S rRNA. All quantitative data, except those in (**s**), represent means ± s.e.m. *P < 0.05; **P < 0.01 (Student’s t-test or one-way ANOVA with Tukey’s post-hoc test).

We next established the STAM-MASH model with MFG-E8 KO mice. We first confirmed that *Mfge8* genotype did not affect the basic features of the disease model including body and tissue weights and blood glucose levels (Supplementary Fig. 3a–c). We then assessed disease severity on the basis of NAS, which is defined as the sum of the scores for three components: steatosis (score of 0–3), lobular inflammation (score of 0–3), and ballooning (score of 0–2). This histological analysis revealed that, among STAM-MASH model mice, NAS was significantly lower in MFG-E8 hetero- and homozygous KO mice than in WT mice (Fig. 2a), with this improvement being due to amelioration of lobular inflammation and ballooning in the KO animals (Fig. 2b–d). Consistent with these histological findings, the amounts of mRNAs for inflammatory cytokines (Fig. 2e–g), the F4/80 macrophage marker (Fig. 2h), and type I collagen (Fig. 2i) in the liver were significantly lower in the KO mice. Serum levels of liver enzymes were also lower in the KO animals (Fig. 2j, k), reflecting attenuation of inflammation and hepatocyte damage, whereas the serum concentrations of triglyceride (TG) and total cholesterol as well as hepatic TG content were not affected by genotype (Supplementary Fig. 3d–f). On the other hand, we also confirmed that inflammatory gene expression in eWAT and cytokine levels in the blood were downregulated in the KO animals (Supplementary Fig. 3g, h).

Immunohistochemical staining revealed that, among the STAM-MASH model animals, the numbers of F4/80^+^ cells and hCLSs in the liver were greatly reduced for MFG-E8 KO mice relative to WT mice (Fig. 2l–o). Sirius red staining also showed a marked reduction in the extent of hepatic fibrosis in the KO mice (Fig. 2p–r). Given that a recent meta-analysis revealed that hepatic fibrosis was associated with overall mortality in individuals with MASLD^40^, we performed a survival analysis for the STAM-MASH model animals. The STAM model differs markedly from diet-induced MASH models in both the kinetics of disease progression and the rate of tumor development. Following neonatal streptozotocin (STZ) administration and high-fat feeding, STAM mice exhibit rapid, synchronized progression to steatohepatitis and fibrosis by 8–10 weeks of age, and develop multiple liver tumors by 16–20 weeks^41^. Consequently, unlike western diet or high-fat/high-fructose diet models, where spontaneous hepatocellular carcinoma (HCC) typically emerges after 40–60 weeks^42^, substantial proportion of STAM mice die within approximately 3–4 months. Kaplan-Meier analysis showed that the survival time of STAM-MASH WT mice was significantly shorter than that for *Mfge8* homozygous KO mice, with mean values of 65.24 ± 4.46 and 85.45 ± 5.77 days after the onset of HFD feeding, respectively (Fig. 2s, t). Liver-related events such as HCC or liver failure are generally considered the predominant causes of mortality in the STAM system, however, we did not systematically determine the cause of death for each mouse. Thus, the potential contribution of systemic metabolic deterioration or extrahepatic complications cannot be fully excluded and represents a limitation of this analysis.

The STAM-MASH model is generated in part by HFD feeding, which induces adipocyte hypertrophy as well as *Mfge8* expression in adipose tissue (Fig. 1a, Supplementary Fig. 2a–c). To further examine the role of adipose-derived MFG-E8 in MASH pathogenesis, we assessed the effects of MFG-E8 on disease activity in a methionine-restricted choline-deficient diet (MRCD)-fed mice, a well-established nonobese MASH model^43^. As expected, induction of *Mfge8* expression in adipose tissue and an increase in the circulating concentration of MFG-E8 were not observed in MRCD-fed WT mice (Supplementary Fig. 4a–d). Histological analysis revealed that NAS for MRCD-fed WT mice (5.67 ± 0.48) was similar to that for STAM-MASH WT mice (5.57 ± 0.32) (Fig. 2a, Supplementary Fig. 4e). However, in contrast to the STAM-MASH model, NAS for MRCD-fed mice did not differ between WT and MFG-E8 KO animals (Supplementary Fig. 4e–h). Similarly, the expression of inflammatory genes in the liver as well as the serum levels of liver enzymes did not differ between WT and KO mice of the MRCD model (Supplementary Fig. 4i, j). Although this analysis is limited by the absence of direct histological quantification of fibrosis (such as Sirius Red staining), the comparable mRNA expression levels of key fibrosis-related genes support the interpretation that *Mfge8* deficiency does not alter fibrotic progression under MRCD feeding. Nonetheless, the lack of histological confirmation remains an important limitation that should be addressed in future studies.

Together, these findings revealed that there was no effect of MFG-E8 deficiency on disease activity in MRCD-fed mice, suggesting that the expression of MFG-E8 in adipose tissue is important in the pathogenesis of obesity-associated MASH.

### Effects of MFG-E8–containing EV injection in MFG-E8 KO mice

To further validate the importance of MFG-E8 in MASH pathogenesis, we examined the effects of MFG-E8 reconstitution in STZ-treated MFG-E8 KO mice by daily intraperitoneal injection of MFG-E8–containing EVs for 1 week beginning at 4 weeks of age. While adipose tissue likely represents a major physiological source of MFG-E8 in obesity, we utilized macrophage-derived EVs because of the technical challenges in obtaining sufficient and reproducible EVs from primary adipocytes. We first confirmed that *Mfge8* genotype had no effect on the size distribution or mean size of EVs isolated from conditioned medium of cultured peritoneal macrophages (Supplementary Fig. 5a–c).

**Fig. 3:**
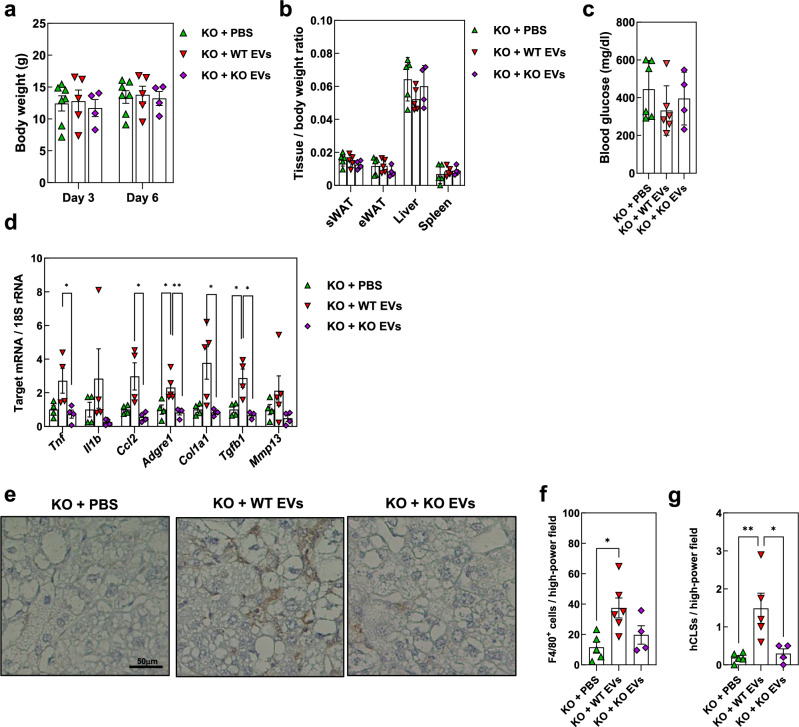
Effects of EV administration on hepatic inflammation and histology in neonatally STZ-treated MFG-E8–deficient mice. **a** Body weight of streptozotocin (STZ)-treated MFG-E8 KO mice (*n* = 4–7) injected daily for 3 or 6 days with PBS or extracellular vesicles (EVs). **b** Tissue-to-body weight ratios of sWAT, eWAT, liver, and spleen (*n* = 4–7). **c** Blood glucose concentrations (*n* = 4–7). **d** RT–qPCR analysis of *Tnf*, *Il1b*, *Ccl2*, *Adgre1*, *Col1a1*, *Tgfb1*, and *Mmp13* mRNA abundance in liver (*n* = 4–7). **e** Representative immunohistochemical staining of F4/80 in liver of STZ-treated MFG-E8 KO mice injected daily for 7 days with PBS (KO + PBS), EVs derived from WT mice (KO + WT EVs), or EVs derived from MFG-E8 KO mice (KO + KO EVs). **f** Quantification of F4/80-positive cells per high-power field (0.2 mm²) in liver. **g** Quantification of hCLS per high-power field (0.2 mm²) in liver (*n* = 4–6). Throughout Fig. 3, marker shapes and colors indicate the treatment administered to STZ-injected MFG-E8–knockout mice: green triangles, PBS; red inverted triangles, EVs derived from wild-type mice; purple diamonds, EVs derived from MFG-E8 KO mice. PBS or EVs were injected once daily for 7 days, except in panel a, where injections were performed for 3 or 6 days as indicated. The amounts of mRNAs were normalized to 18S rRNA. All quantitative data represent means ± s.e.m. *P < 0.05; **P < 0.01 (one-way ANOVA with Tukey’s post-hoc test).

No differences in body weight (Fig. 3a), tissue/body weight ratios (Fig. 3b), or blood glucose levels (Fig. 3c) were apparent among STZ-treated MFG-E8 KO mice during or after repeated injection of EVs from WT or MFG-E8 KO mice or of PBS as a vehicle control. RT–qPCR analysis revealed that injection of WT EVs increased inflammatory and fibrosis-related gene expression in the liver, whereas that of EVs from KO mice had no such effects (Fig. 3d). Injection of EVs from WT or MFG-E8 KO mice did not influence such gene expression in the spleen, sWAT, or eWAT (Supplementary Fig. 5d–f), indicative of a selective action of the WT EVs in the liver. Consistent with these results, the numbers of F4/80^+^ cells and hCLSs in the liver were significantly increased by injection of WT EVs but not by that of the KO EVs (Fig. 3e–g).

The administration period of MFG-E8-containing EVs in the rescue experiment was limited to one week, suggesting that histological fibrosis had not yet been established at the time of analysis. Although the changes in hepatic mRNA expression of *Col1a1*, *Mmp13*, and *Tgfb1* were observed in this study, future studies involving longer-term supplementation and morphological assessment (such as Sirius red staining) will be required.

Together, these findings suggested that circulating MFG-E8 selectively promotes hepatic inflammation and fibrogenic reactions during MASH development.

### Inflammatory gene expression in macrophages cocultured with apoptotic hepatocytes

To examine the role of MFG-E8 in macrophage-hepatocyte interaction, we cocultured peritoneal macrophages derived from MFG-E8 KO mice with Hepa1c1c7 cells in which the endogenous *Mfge8* gene had been deleted with the use of the CRISPR/Cas9 system. In similar past co-culture experiments, dead cells and phagocytic cells were co-cultured for over ten hours prior to evaluating inflammation, concluding that the phagocytosis process was anti-inflammatory^44,45^. In our co-culture experiments, we aimed to evaluate direct interactions between dead cells and macrophages independent of MFG-E8-regulated phagocytosis, and assessed the inflammatory response after 3 h.

**Fig. 4:**
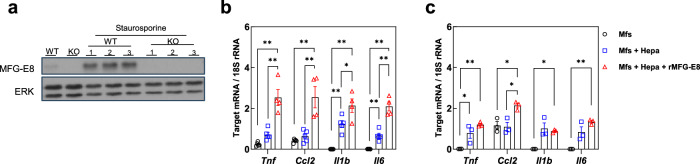
Effects of apoptotic hepatocytes and rMFG-E8 on inflammatory gene expression in MFG-E8 KO macrophages. **a** Immunoblot analysis of MFG-E8 and ERK (loading control) in parental wild-type (WT) Hepa1c1c7 cells and Hepa1c1c7 cells in which endogenous *Mfge8* was deleted using the CRISPR–Cas9 system. Cells were exposed to staurosporine to induce apoptosis (three replicates shown), a known stimulus for MFG-E8 expression. **b** RT–qPCR analysis of *Tnf*, *Ccl2*, *Il1b*, and *Il6* mRNA abundance in thioglycolate-elicited peritoneal macrophages (*n* = 4–5) cocultured directly with apoptotic *Mfge8*-deficient Hepa1c1c7 cells. **c** RT–qPCR analysis of *Tnf*, *Ccl2*, *Il1b*, and *Il6* mRNA abundance in thioglycolate-elicited peritoneal macrophages (*n* = 3) cocultured with apoptotic *Mfge8*-deficient Hepa1c1c7 cells across a Transwell membrane. In (**b**, **c**), apoptosis was induced in Hepa1c1c7 cells by staurosporine treatment. The apoptotic hepatocytes were cocultured with macrophages for 3 h under the indicated conditions in the presence or absence of recombinant MFG-E8 protein (rMFG-E8; 1.0 µg/ml). Cells were then harvested for RT–qPCR analysis. Marker shapes and colors in the bar graphs indicate macrophage culture conditions: black circles, macrophages alone (Mfs); blue squares, macrophages cocultured with dead hepatocytes (Mfs + Hepa); red triangles, macrophages cocultured with dead hepatocytes in the presence of rMFG-E8 (Mfs + Hepa + rMFG-E8). The amounts of mRNAs were normalized to 18S rRNA. Data are means ± s.e.m. *P < 0.05, **P < 0.01 (one-way ANOVA and Tukey’s post hoc test).

To verify the knockout efficiency of *Mfge8*, we evaluated its expression in genome-edited hepatocytes following apoptosis induction, a known stimulus that induces MFG-E8 expression^46^. In wild-type hepatocytes, MFG-E8 protein was markedly upregulated after staurosporine treatment (100 nM), whereas this induction was completely abolished in *Mfge8*-KO cells, confirming the effective disruption of *Mfge8* (Fig. 4a). The percentage of CD11b^+^F4/80^+^ macrophages isolated from MFG-E8 KO mice was similar to that isolated from WT mice (Supplementary Fig. 6a–c), and no obvious difference in M1- and M2-like marker or inflammatory cytokine gene expression was apparent between cells of the two genotypes (Supplementary Fig. 6d–g). We induced apoptosis in the MFG-E8–deficient Hepa1c1c7 cells by exposing them to staurosporine (100 nM) and then cocultured them with MFG-E8 KO macrophages for 3 h in the absence or presence of recombinant MFG-E8 protein (rMFG-E8). Unlike in vivo rescue experiments, we used recombinant MFG-E8 to precisely control concentration and timing of stimulation. Since MFG-E8 protein exhibits high reactivity with PS, the added MFG-E8 protein is thought to rapidly react and bind to PS-expressing EVs derived from cultured cells. Therefore, it is considered that there is little difference compared to EV-bound form.

RT–qPCR analysis revealed that the apoptotic cells induced inflammatory cytokine gene expression in the macrophages and that this effect was enhanced in the presence of rMFG-E8 (Fig. 4b). Finally, we performed a coculture experiment under conditions in which the two cell types were separated by a Transwell membrane, so that they shared the same medium but were not in direct contact with each other. In this instance, the apoptotic cells again induced inflammatory gene expression in the macrophages but the stimulatory effect of rMFG-E8 supplementation was greatly attenuated (Fig. 4c).

MFG-E8 has been reported to exert anti-inflammatory effects on macrophages through STAT3 activation when applied alone^47^. In contrast, our data show that rMFG-E8 enhances inflammatory gene expression only in the presence of apoptotic hepatocytes, and this effect is markedly reduced when physical contact is prevented. These findings highlight that the functional properties of MFG-E8 is context-dependent and that its pro-inflammatory activity in MASH could be closely linked to its role in apoptotic cell recognition and processing.

## Discussion

Visceral adiposity correlates with hepatic inflammation and fibrosis during MASH development^7^, implicating adipose-liver cross-talk in MASH pathogenesis. We have now identified an adipose tissue–derived secreted protein, MFG-E8, as a candidate mediator of the link between visceral fat and hepatic inflammation and fibrosis.

We found that most inflammatory markers were greatly suppressed in MASH model mice established on the MFG-E8–null background. MFG-E8 was previously shown to promote the absorption of dietary fat and the transport of fatty acids into cells, with MFG-E8 KO mice thus being found to be resistant to weight gain and the development of hepatic steatosis and metabolic disorders when fed a HFD^48^. The effects of MFG-E8 deficiency on body composition were most apparent when mice were maintained on the HFD for at least 12 weeks. In the present study, we mostly studied MFG-E8 KO mice that had been maintained on a HFD for only 4 weeks, at which time body and tissue weight, hepatic lipid accumulation, and serum lipid parameters did not differ between the KO and WT animals. Our data therefore indicate that MFG-E8 promotes hepatic inflammation, fibrosis, and MASH progression independently of lipid metabolism.

Suppression of proinflammatory gene expression by *Mfge8* ablation was also apparent in eWAT, the main site of MFG-E8 production, in the STAM-MASH model (Supplementary Fig. 3g). This finding suggests that MFG-E8 might activate local immune cells in adipose tissue and thereby give rise to increased circulating cytokine levels (Supplementary Fig. 3h), which may have a secondary adverse effect on MASH disease. In addition, it is possible that a localized increase in hepatic MFG-E8 expression contributes to MASH pathogenesis (Supplementary Fig. 2g). While MFG-E8 is classically regarded as a phagocyte-derived opsonin, our data indicate that hepatocytes themselves can upregulate MFG-E8 upon apoptotic stress (Fig. 4a). Such localized induction may act as a self-directed “eat-me” cue, enhancing efferocytosis and shaping the inflammatory microenvironment during MASH progression. Although we could not rule out these possibilities, administration of MFG-E8–containing EVs in MFG-E8–deficient STAM-MASH mice abolished the protective effects of *Mfge8* deletion apparent in the liver without induction of proinflammatory gene expression in other abdominal organs including eWAT. Furthermore, in the nonobese, MRCD-fed MASH model, in which MFG-E8 was not induced in adipose tissue, no differences in the examined pathological parameters were detected between WT and MFG-E8 KO mice. Together, these findings suggest that MFG-E8 expression in adipose tissue is important for the progression of obesity-associated MASH pathology, and that MFG-E8 reaches the liver via the circulation and directly contributes to the development of liver inflammation and fibrosis.

MFG-E8 protects against inflammatory reactions and excessive tissue fibrosis by facilitating the removal of apoptotic cells and ECM clearance by dendritic cells and macrophages^49^. However, it also exerts a proinflammatory action through accumulation of its cleaved fragment, medin, an amyloidogenic factor^50^. Moreover, an RGD domain at the NH_2_-terminus of the protein has been found to activate TGF-β signaling and to increase ECM production by binding integrins on hepatic stellate cells, leading to tissue fibrosis^51,52^. Our current findings further suggest that MFG-E8 may exhibit its proinflammatory effect by regulating interactions between macrophages and dying hepatocytes. Our in vitro coculture experiments revealed that the induction of inflammatory responses in macrophages by MFG-E8 required direct contact of the macrophages with dead hepatocytes. Knockout of MFG-E8 abolished the formation of hCLSs associated with MASH, and this effect was reversed by administration of exogenous MFG-E8–containing EVs. These results support the idea that MFG-E8 facilitates the aggregation of macrophages around apoptotic hepatocytes, a process that may contribute to inflammatory signaling in the MASH liver. Importantly, no obvious tissue-structure abnormalities were observed in *Mfge8*-deficient mice, suggesting that global efferocytic capacity remains largely intact, possibly through PS-recognition pathways independent of MFG-E8 (e.g., MerTK, Stabilin-2, TIM4)^53^. Taken together, our data and previous studies indicate that MFG-E8 may exert dual and context-dependent functions; (1) An inflammatory role, in which MFG-E8 enhances macrophage clustering around apoptotic hepatocytes (hCLS formation) and amplifies dead-cell–derived inflammatory cues, and (2) an anti-inflammatory role via its efferocytic effect, in which MFG-E8 promotes efficient clearance of apoptotic cells^54^. Which of these two functions predominates may depend on factors such as the tissue source and abundance of MFG-E8, the metabolic background, and the stage of disease. In the context of obesity, where adipose-derived MFG-E8 is markedly elevated, the pro-inflammatory, macrophage-clustering function appears to dominate, consistent with recent reports showing that adipose-tissue EVs can drive hepatic inflammation and fibrosis^55^. Direct in vivo measurements of apoptotic cell burden in NASH model (wild-type and MFG-E8 knockout) will provide further insight to definitively determine how MFG-E8 loss affects net clearance efficiency.

Expression of the MFG-E8 gene is regulated by transcription factors including Sp1 and AP-1^56^as well as by microRNAs^57^. Although we did not investigate the underlying molecular mechanisms, we confirmed that cell death in hepatocytes and hypertrophy of adipocytes were accompanied by increased expression of MFG-E8. *Mfge8* expression in adipose tissue was upregulated to a markedly greater extent in the STAM-MASH model than in a simple obesity model, even though the size of adipocytes was smaller in the former than in the latter model. This difference is thought to result from impaired development of adipose tissue and the insulin resistance due to insulin deficiency induced by STZ administration after birth, and from the subsequent complete depletion of insulin in adulthood^58^. Insulin signaling is important for the survival of adipocytes in HFD-fed mice^59^, and the state of insulin dysfunction in STAM-MASH mice may adversely affect the survival of adipocytes and contribute to the induction of MFG-E8 expression.

As with most experimental studies, this work has several limitations.

First, peritoneal macrophages were used for in vitro assays and as the source of EVs for the rescue experiments. Although they offer technical consistency and low baseline activation, their properties are not identical to hepatic macrophages or adipocytes. EVs from wild-type and *Mfge8*-deficient macrophages were comparable in size and distribution, but because the biological effects of MFG-E8 may differ depending on cellular or tissue origin, future studies using hepatic macrophages, adipocyte-derived EVs, or improved methods for their isolation, will be important.

Second, previous studies have reported protective roles of hepatic MFG-E8 through ASK1- and TLR4/NF-κB–related pathways^60,61^, whereas our findings implicate adipose-derived, EV-associated MFG-E8 in promoting macrophage activation and hCLS formation. These differences likely reflect distinct tissue sources and metabolic backgrounds; therefore, adipose-specific perturbation of MFG-E8 will be required to mechanistically define tissue-dependent functions.

Finally, the STAM model was chosen for its reproducible and synchronized progression, which allowed us to evaluate adipose–liver crosstalk within a defined timeframe. However, it does not fully replicate the metabolic features of human MASH. Validation in metabolically driven diet-induced models will strengthen translational relevance, although such models present technical challenges in *Mfge8*–deficient mice due to their resistance to long-term diet-induced metabolic dysregulation.

Although no data are available regarding the expression and circulating concentration of MFG-E8 in individuals with MASH, serum levels of MFG-E8 have been shown to be increased in obese or diabetic individuals^28,62^. However, given that MFG-E8 is thought to play an important role in tissue repair through efficient removal of dead cells, it may contribute to optimal tissue homeostasis, and its deficiency has been found to worsen certain diseases^63,64^. Caution is therefore warranted with regard to development of MFG-E8 as a target for MASH therapy or prevention.

## Methods

### Cell culture and treatment

3T3-L1 preadipocytes were kindly provided by T. Hosaka (University of Shizuoka, Japan). The cells were maintained under 7.5% CO_2_ at 37 °C in high-glucose Dulbecco’s modified Eagle’s medium (DMEM, Sigma) supplemented with 10% calf serum (Thermo Fisher Scientific) and penicillin-streptomycin (100 U/ml, Thermo Fisher Scientific). For adipogenic differentiation, cells maintained at confluence for 48 h were exposed for 48 h to high-glucose DMEM supplemented with 10% fetal bovine serum (FBS, GE Healthcare), penicillin-streptomycin (100 U/ml), insulin (10 μg/ml, Fujifilm Wako), 500 μM isobutyl methylxanthine (Sigma), 1 μM dexamethasone (Fujifilm Wako), and 1 μM troglitazone (Cayman Chemical). The medium was then changed to high-glucose DMEM supplemented with 10% FBS and penicillin-streptomycin (100 U/ml) and refreshed daily thereafter. Cells were harvested for reverse transcription and quantitative polymerase chain reaction (RT-qPCR) analysis as described below.

Hepa1c1c7 murine hepatoma cells were maintained under 5.0% CO_2_ at 37 °C in low-glucose DMEM (Sigma) supplemented with 10% FBS and penicillin-streptomycin (100 U/ml). For deletion of the MFG-E8 gene with the CRISPR/Cas9 system, the DNA sequence encoding the guide RNA (5′-TCTGGCCTCTTCGCCGCGTC-3′) was inserted into the pSpCas9(BB)-2A-Puro (PX459) vector (Addgene), and the resulting plasmid was introduced into Hepa1c1c7 cells by transfection with the use of the Lipofectamine 2000 reagent (Invitrogen). The cells were then subjected to selection by culture in the presence of puromycin (2 μg/ml) before repetition of the transfection and selection processes to ensure efficient gene deletion. For induction of apoptosis, cells were treated with 100 nM staurosporine (Sigma) in serum-free medium for 2 h and then replaced fresh serum free medium and cultured for 24 h. They were then cocultured with macrophages prepared as described below.

For in vitro coculture experiment and EV isolation, peritoneal macrophages were isolated from 10 to 15-week-old female mice fed a standard diet. 4 days after injection of 2 ml/animal of 4% thioglycolate in Ca^2+^- and Mg^2+^-free phosphate-buffered saline (PBS(–)) into the abdominal cavity, the elicited peritoneal cells were collected by peritoneal lavage with 5 mL/animal of DMEM (Sigma) from the peritoneal cavity. Collected cells were spined down by centrifuge at 400 × *g* for 5 min at 4 °C, resuspended in supplemented DMEM, and seeded on culture plated at appropriate density.

For coculture experiments, peritoneal macrophages were seeded on 24 well plate (2.0 × 10^6^ cells/ well) and cultured for 24 h prior to the coculture. Apoptotic Hepa1c1c7 cells were incubated with the macrophages for 3 h (2.5 × 10^4^ cells/ well), with or without separation by a 0.4 mm Transwell membrane filter. Recombinant MFG-E8 (R&D systems) were added to culture medium at 1.0 ug/ml together with He1c1c7 cells.

### Flow Cytometry

Seeded macrophages were washed with PBS(–) three times, incubated in 2 mM EDTA / PBS(–) for 10 minutes, and detached from culture dish by pipetting. The collected cells were then resuspended and incubated in 2% FBS / PBS(–) containing 0.5 μg/ml anti-mouse CD16 / CD32 (Fc Shield) (2.4G2) (Cytek) for 10 minutes to prevent unwanted binding of antibodies to FcR. FITC-labeled anti-mouse F4/80 antibody (Biolegend) and PE-labeled anti-mouse CD11b antibody (Biolegend) were subsequently added to the mixture and incubated in ice for another 30 minutes. After washing the cells with 2% FBS / PBS(–) ‘ two times, the cells were suspended in 2% FBS / PBS(–) with 1 mg/ml 7-AAD (7-aminoactinomycin D) and analyzed with BD FACSVerse instrument (Becton Dickinson).

### Animals and MASH models

Animal studies were performed according to the guidelines of and approved by the Animal Research Committee of Tokushima University (approval code: T2020-75). All procedures complied with institutional and national regulations for animal care and use. All mice were monitored at least once daily for general health and signs of distress. All mice were housed in a specific pathogen–free (SPF) facility in a room with a controlled temperature (22 ± 2°C), humidity (40–60%), and a 12 h-light, 12 h-dark cycle, and they were provided ad libitum access to tap water and food (MFG, Oriental Yeast). All mice were acclimatized to the housing environment for at least one week before the initiation of any experimental procedures.

MFG-E8 knockout (KO) mice^65^ (RBRC01726) were obtained from RIKEN BRC (Tsukuba, Japan), and maintained on C57BL/6 background. C57/B6 mice were purchased from Charles River Laboratories (Wilmington, MA). STAM-MASH model mice were generated by subcutaneous injection of 200 µg of streptozotocin (STZ in PBS(-), Sigma) at 48 h after birth and maintenance on an HFD (HFD60, Oriental Yeast) starting at 4 weeks of age^66^. The mice were studied at 8 weeks of age unless noted otherwise. For experiments requiring younger animals, 5–6-week-old mice were used. The age in these experiments is explicitly indicated in the legend of the corresponding figure. Another MASH model was generated by feeding mice with a methionine-restricted choline-deficient diet (MRCD, Research Diets) from 8 to 12 or 16 weeks of age. These mice were carefully monitored and were killed by CO_2_ asphyxiation followed by cervical dislocation if they experienced excessive weight loss (> 20% in 1 week) or showed signs of distress. Mice fed with normal chow (NC) (MFG, Oriental Yeast) were used as control.

Animals were randomly assigned to experimental groups. Investigators performing histological evaluation and quantitative analysis were blinded to genotypes and treatments. Sample sizes were determined based on previous studies using similar models. No statistical method was used to determine sample size. The experimental unit in all animal experiments was a individual mouse. Each mouse was treated and analyzed independently, and sample number reported in the figure legends refers to the number of individual animals.

For tissue collection and blood sampling, mice were anesthetized with a mixture of medetomidine (0.3 mg/kg), midazolam (4 mg/kg), and butorphanol (5 mg/kg). For bone marrow transplantation, isoflurane inhalation anesthesia was used during the tail-vein injection of donor bone marrow cells.

Serum samples collected from mice were analyzed for clinical markers by Nagahama Life Science Laboratory (Oriental Yeast). The blood glucose level was measured by Medisafe FIT pro (Terumo, Japan). To determine hepatic TG content, dissected liver tissue was lysed in the buffer consisting of 10 mM Tris-HCl (pH 7.4), 1 mM EDTA, and 0.1% TritonX-100. The debris was precipitated by centrifugation and the resulting supernatant fraction was used to measure TG according to Kit’s instructions (L-type Triglyceride M) (Fujifilm Wako). For enzymatic dissociation, dissected epididymal white adipose tissue (eWAT) was cut into small pieces and digested by incubation for 45 min at 37°C with gentle shaking in PBS(–) containing type II collagenase (2500 U/ml, Worthington) and 2% bovine serum albumin (Sigma). The digested material was passed through a 250-μm nylon mesh, and the filtrate was centrifuged at 400 × *g* for 5 min at 4°C. Floating cells were collected as adipocytes, and the pellet as the stromal vascular fraction (SVF).

For bone marrow transplantation, mice were exposed to 9.0 Gy of x-radiation at 5 weeks of age. On the next day, bone marrow cells (5 × 10^5^ cells per gram of body weight) isolated from the hindlimbs of donor mice were injected into the irradiated mice through the tail vein under brief isoflurane anesthesia, and the recipients were maintained on an NC diet for 3 weeks before feeding with an HFD for up to 22 weeks.

All experiments included appropriate control groups (e.g., NC-fed mice, wild-type mice, and PBS-treated mice), and the groups being compared are specified in each figure legend.

### RNA isolation and RT-qPCR analysis

Total RNA was isolated from tissues or cultured cells with the use of the RNAiso Plus reagent (Takara) and was subjected to RT with the use of a PrimeScript RT Reagent Kit (Takara). The resulting cDNA was subjected to qPCR analysis with SYBR Green Master Mix (Applied Biosystems) and specific primers (Supplementary Table 1) in a StepOnePlus instrument (Applied Biosystems). The amount of each target mRNA was normalized by that of 18S rRNA.

### Isolation, measurement, and in vivo administration of EVs

Peritoneal macrophages were incubated in serum-free medium (DMEM) (Sigma) for 48 h, after which extracellular vesicles (EVs) were purified from the conditioned medium by size-exclusion chromatography with a qEV column (Izon) and automatic fraction collector (Izon). EV particle size and number were determined with a qNano system (Izon), and EVs (6 × 10^4^) were injected intraperitoneally once a day for 1 week in 4-week-old MFG-E8 KO mice that had received a neonatal injection of STZ.

For EV isolation from serum, serum samples were first centrifuged at 15,000 × *g* for 5 min at 4 °C to remove debris and the supernatants were then further centrifuged at 100,000 × *g* for 60 min at 4 °C. The final pellets were washed twice with ice-cold PBS(–).

### Preparation of cell or tissue lysates and immunoblot analysis

Cultured cells were washed with ice-cold PBS(–) and lysed by vigorous agitation in RIPA buffer (20 mM Tris-HCl [pH 7.4], 150 mM NaCl, 1% sodium deoxycholate, 0.1% SDS, 1% Nonidet P-40, 2 mM EDTA) supplemented with 1 mM phenylmethylsulfonyl fluoride (Sigma). Frozen tissues were disrupted with a Dounce homogenizer in RIPA buffer. Cell and tissue lysates were centrifuged at 15,000 × *g* for 20 min at 4 °C to remove debris, and the resulting supernatants were collected for analysis. EV samples for immunoblot analysis were prepared by suspension of the washed pellet obtained by ultracentrifugation as described above in RIPA buffer. Protein concentration was determined with a Pierce BCA Protein Assay Kit (Thermo Fisher Scientific).

Samples for immunoblot analysis were fractionated by SDS-polyacrylamide gel electrophoresis, the separated proteins were transferred to a polyvinylidene difluoride membrane (Millipore), and the membrane was incubated first for 16 h at 4 °C with primary antibodies diluted 1/500 to 1/2000 in Tris-buffered saline containing 0.1% Tween 20 and then for 1 h at room temperature with horseradish peroxidase–conjugated secondary antibodies to goat (Zymed) or rabbit (MBL) immunoglobulin G. The primary antibodies included those to mouse MFG-E8 (AF2805, R&D Systems), to mouse CD9 (20597-1-AP, Proteintech), and to mouse ERK (#9101, Cell Signaling). The membrane was then exposed to Clarity Western ECL Substrate (Bio-Rad), and signals were detected with X-ray film (Fujifilm Wako) and an X-ray film processor (FPM100, Fujifilm Wako). Band intensity was determined with the use of ImageJ software.

### Histological analysis

Dissected tissues were fixed with neutral buffered formalin and embedded in paraffin. Paraffin blocks were cut into sections with a thickness of 3 to 5 μm. Liver sections were stained with hematoxylin-eosin for determination of the NAFLD (MASLD) activity score (NAS)^67^ and were stained with Sirius red (0.1% Direct Red, Sigma) and Van Gieson Solution P (Fujifilm Wako) for assessment of fibrosis. For NAS scoring, three randomly selected images of the liver (zone 3) were examined and averaged for each mouse.

For immunohistochemistry, tissue sections were exposed to proteinase K (1 mg/ml, Fujifilm Wako) and then to 0.03% H_2_O_2_ in PBS(–) for antigen retrieval and inactivation of endogenous peroxidases, respectively. The sections were then incubated with antibodies to F4/80 (diluted 1/200) for 16 h at 4 °C. Immune complexes were detected with horseradish peroxidase–conjugated secondary antibodies to rat immunoglobulin G (1/200 dilution, Kirkegaard & Perry) and 3,3′-diaminobenzidine (Fujifilm Wako).

Quantitative histological analysis was conducted in a blinded manner.

### Measurement of adipocyte size

Dissected eWAT was fixed by exposure to osmium tetroxide at 37 °C for 3 days with agitation. Dissociated cells from the adipose tissue were passed through a 250 μm nylon mesh and then subjected to filtration with a 25 μm mesh, and the size of the cells trapped on the mesh was measured. The cells were suspended in Isoton II Dilutant (Beckman), and cell size was measured with a Coulter Multisizer 3 device (Beckman).

### DNA microarray analysis

Total RNA was purified from mouse eWAT with the use of the Trizol reagent (Thermo Fisher Scientific), and in vitro transcription was performed with a Message Amp kit (Ambion) according to the protocols for cRNA amplification and biotin labeling. A hybridization cocktail was prepared as described in the Affymetrix manual for the MU74V2 gene chip, and this cocktail was then subjected to hybridization by incubation overnight with the probe array. Hybridized samples were scanned with a 3000 GeneChip Scanner (Affymetrix), and the scanned images were analyzed with Affymetrix GeneChip Analysis Software (GCOS). The fold differences in hybridization intensity between samples from adipose tissue of normal chow-fed and high fat-fed mouse were determined.

### Single cell RNA sequence reanalysis

Public mouse single-cell RNA-seq datasets were obtained from the CELLxGENE portal, including liver (GSE166504)^38^ adipose tissue (GSE176067)^39^. Data were downloaded as. h5ad files and analyzed in Python (Google Colab). AnnData were processed using Scanpy (v1.10). Library-size normalization (sc.pp.normalize_total(target_sum = 1e4)) followed by log-transformation (sc.pp.log1p) was applied. Analyses were performed using Python 3.x, Scanpy 1.10, anndata 0.x on Google Colab.

### Statistical analysis

Quantitative data are presented as the mean ± s.e.m. Differences between or among groups were assessed by Student’s *t*-test or by one-way analysis of variance (ANOVA) followed by Tukey’s post hoc test. Overall survival was evaluated by the Kaplan-Meier method and log-rank test. All statistical analyses, including normality assessment (Shapiro–Wilk), were performed with GraphPad Prism ver. 10.4.1. A *P*-value of < 0.05 was considered statistically significant.

No animals, experimental units, or data points were excluded from any analysis.

## Supplementary information

Supplementary information

## Data Availability

Source data are provided with this paper. The remaining data are available from this paper and supplementary materials or the corresponding authors upon request. DNA microarray data are accessible through Gene Expression Omnibus (GEO) database (GSE296147).

## Code availability

The Python code used for reanalysis of publicly available single-cell RNA-seq datasets (GSE166504 and GSE176067) is available from the corresponding author upon reasonable request. All analyses were performed in Google Colab using standard packages including scanpy (v1.9), pandas, numpy, and matplotlib. No custom algorithms were developed.
