# Supplementary material for: Adipose tissue–derived MFG-E8 promotes hepatic inflammation and fibrosis through macrophage activation in a mouse MASH model
Source: NPJ Metab Health Dis. 2026 Feb 20;4:8. doi: 10.1038/s44324-026-00099-0 (PMC12923611; doi:10.1038/s44324-026-00099-0)
Supplement: Supplementary file 1 — Supplementary information [file 44324_2026_99_MOESM1_ESM.pdf]

a

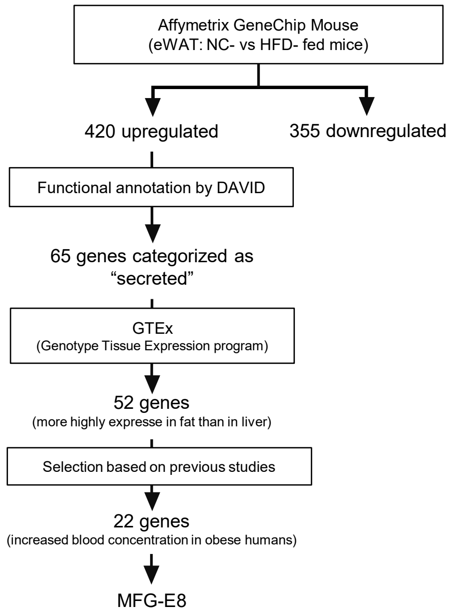

c

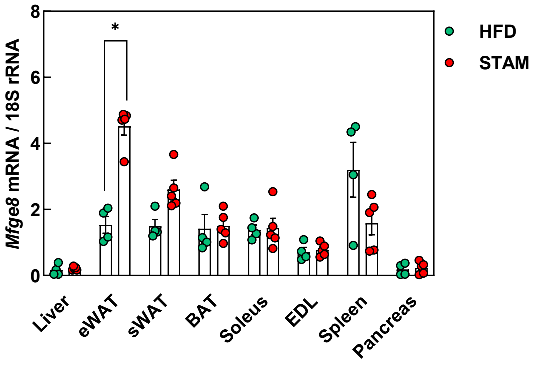

b

ID	Gene name	Accession No	FC (HFD/NC)	TPM ratio (adipose tissue/liver)	Serum level in obesity and/or DM	References
Pm20d1	peptidase M20 domain containing 1	NM_178079	6.96440	10.25	Up	10
Thbs1	thrombospondin 1	NM_001313914	4.59479	27.78	Up	11
S100a8	S100 calcium binding protein A8 (calgranulin A)	NM_013650	4.00000	4.00	Up	12
Fabp5	fatty acid binding protein 5, epidermal	NM_001272097	2.46229	114.74	Up	13
Postn	periostin, osteoblast specific factor	NM_015784	2.29740	77.16	Up	14, 15
Serpinf1	serine (or cysteine) peptidase inhibitor, clade F, member 1	NM_011340	2.14355	1.71	Up	16
Serpine1	serine (or cysteine) peptidase inhibitor, clade E, member 1	NM_008871	2.00000	3.25	Up	17
Ccn1	cellular communication network factor 1	NM_010516	1.86607	8.46	Up	18
Ccn3	cellular communication network factor 3	NM_010930	1.86607	24.43	Up	19, 20
Sema3c	sema domain, immunoglobulin domain (Ig), short basic domain, secreted, (semaphorin) 3C	NM_013657	1.86607	132.89	Up	21
Cilp	cartilage intermediate layer protein, nucleotide pyrophosphohydrolase	NM_173385	1.74110	42.08	Up	22
Fstl1	follistatin-like 1	NM_008047	1.74110	33.60	Up	23
App	amyloid beta precursor protein	NM_007471	1.62450	4.38	Up	24
Anxa1	annexin A1	NM_010730	1.62450	71.57	Up	25
Lox	lysyl oxidase	NM_001286181	1.62450	52.74	Up	26
**Mfge8**	**milk fat globule EGF and factor V/VIII domain containing**	**NM_001045489**	**1.62450**	39.64	Up	27, 28
S100a4	S100 calcium binding protein A4	NM_001410572	1.51572	34.43	Up	29
Anxa2	annexin A2	NM_001409577	1.51572	14.61	Up	30
Dpt	dermatopontin	NM_019759	1.51572	39.08	Up	31
Lgals3	lectin, galactose binding, soluble 3	NM_001145953	1.51572	61.86	Up	32
Lep	leptin	NM_008493	1.51572	2,872.80	Up	33
Sparc	secreted acidic cysteine rich glycoprotein	NM_001290817	1.51572	17.97	Up	34

Supplementary Figure 1 | Identification of MFG-E8 as a potential adipose tissue–derived mediator of MASH progression.
a, Identification workflow for MFG-E8 based on DNA microarray analysis. Microarray analysis was performed with epididymal white adipose tissue (eWAT) of normal chow (NC) or high fat diet (HFD)-fed mice. The 420 genes found to be upregulated (fold change (HFD/NC) > 1.5) in HFD-fed mice were analyzed by DAVID to identify those encoding secreted proteins. Among the resulting 65 genes, those whose expression level in the liver was higher than that in adipose tissue as revealed by the GTEx portal were then excluded. The remaining 52 genes were further narrowed down to 22 genes for which the blood concentration of the encoded protein was found in previous studies to be higher in individuals with obesity or related conditions.
b, List of the 22 selected genes. FC, fold change; TPM, transcripts per million.
c, RT–qPCR analysis of *Mfge8* mRNA abundance in liver, eWAT and subcutaneous white adipose tissue (sWAT), brown adipose tissue (BAT), skeletal muscle (soleus and extensor digitorum longus [EDL]), spleen, and pancreas of HFD-fed mice or STAM-MASH mice ($n = 4$ or 5). Dot colors in the bar graph indicate experimental groups: **green**, HFD-fed mice; **red**, STAM-MASH mice.
The amounts of mRNAs were normalized to 18S rRNA. Data are means ± s.e.m. *$P < 0.05$ (Student's t test).

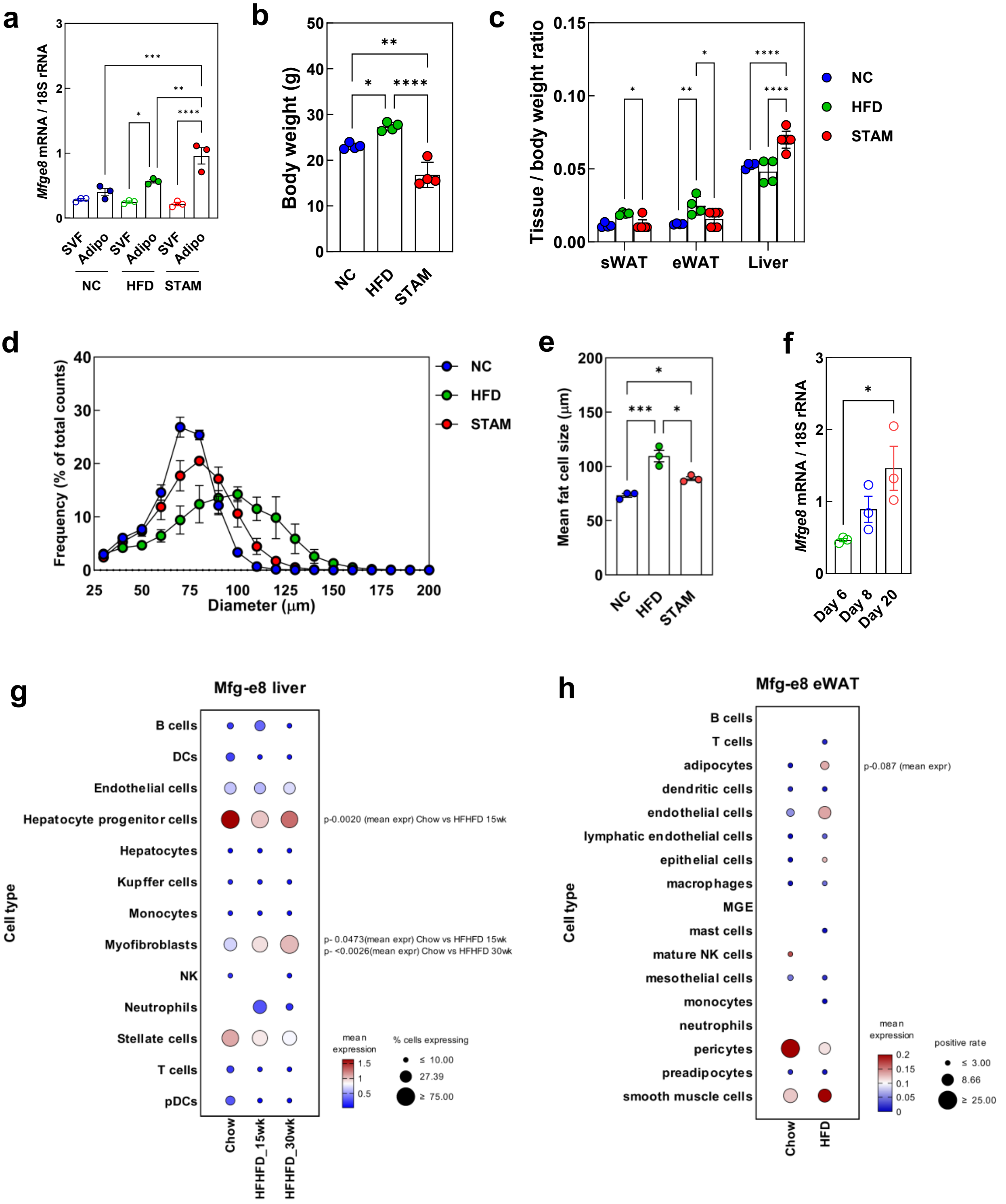

Supplementary Figure 2 | Relation of adipocyte cell size to *Mfge8* expression.
a, RT–qPCR analysis of *Mfge8* mRNA abundance in adipocytes (Adipo) and the stromal vascular fraction (SVF) isolated from eWAT of NC- or HFD-fed mice or of STAM-MASH mice ($n = 3$).
b, Body weight ($n = 4$).
c, Tissue-to-body weight ratio ($n = 4$).
d, Frequency distribution for adipocyte cell diameter in eWAT of mice ($n = 3$).
e, Mean adipocyte cell diameter in eWAT of mice ($n = 3$).
f, RT–qPCR analysis of *Mfge8* mRNA abundance in 3T3-L1 cells at 6, 8, and 20 days after the induction of adipogenic differentiation ($n = 3$ independent experiments).
g, Reanalysis of publicly available single-cell RNA-seq datasets showing *Mfge8* expression across liver (GSE166504) cell types.
h, Reanalysis of publicly available single-cell RNA-seq datasets showing *Mfge8* expression across and adipose tissue (GSE176067) cell types. MGE, mammary gland epithelial cells. Bubble size indicates the proportion of cells expressing *Mfge8*, and color represents the average expression levels.
In panels a–e, marker colors indicate the experimental groups: **blue**, NC-fed mice; **green**, HFD-fed mice; **red**, STAM-MASH mice.
The amounts of mRNAs were normalized to 18S rRNA. All data are means ± s.e.m. *$P < 0.05$, **$P < 0.01$, ***$P < 0.001$, ****$P < 0.0001$ (ANOVA and Tukey's post hoc test). *P* values are shown only when < 0.10 in panel g and h.

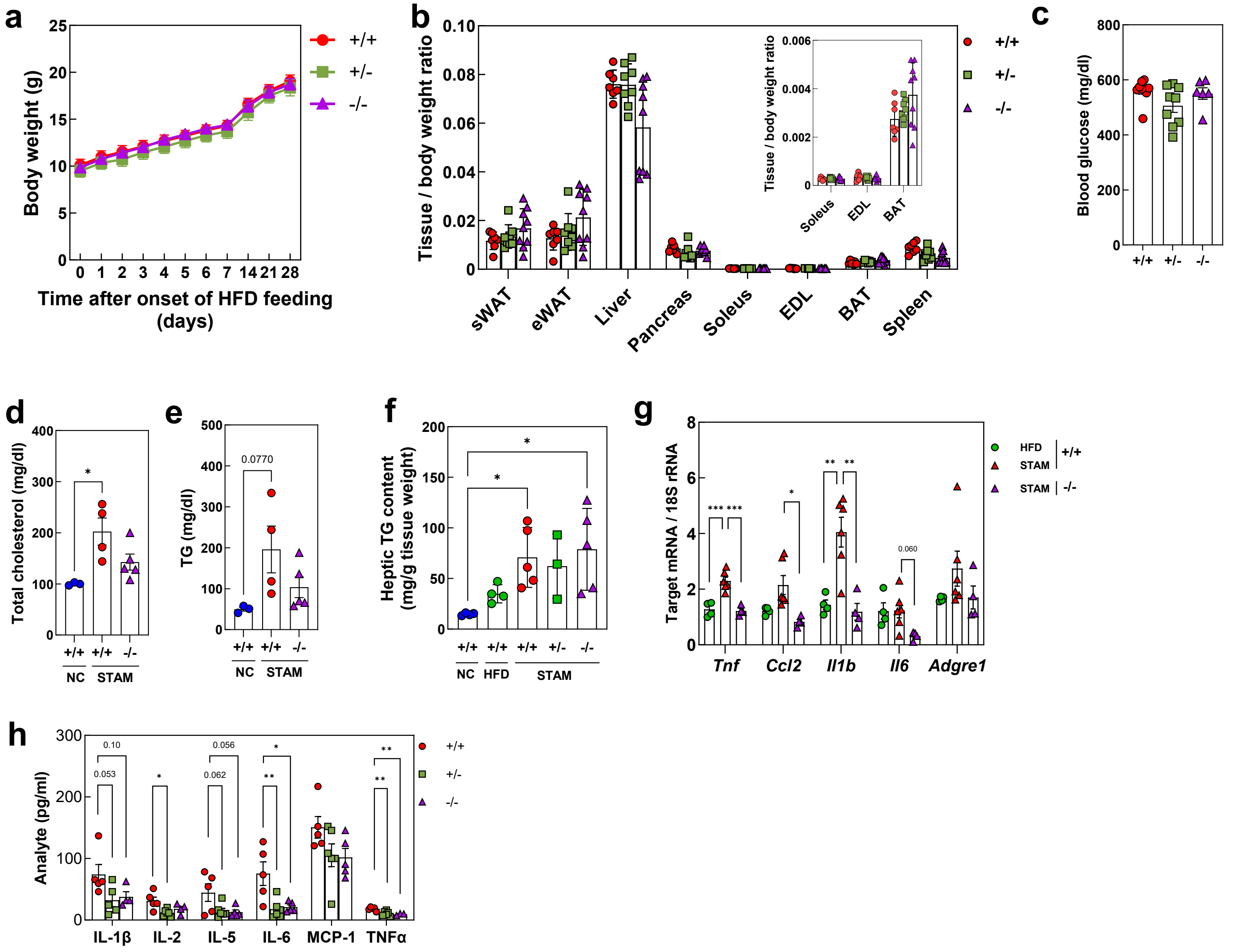

Supplementary Figure 3 | Basic characteristics of STAM-MASH model mice.
a, Body weight of STAM-MASH model (n = 6–11) at the indicated times after the onset of HFD feeding. Line colors indicate the genotype: **red**, wild type mice (WT, +/+) ; **green**, MFG-E8 heterozygous knockout mice (+/–); **purple,** homozygous knockout mice (–/–).
b, Tissue-to-body weight ratios (n = 7–9).
c, Blood glucose concentration (n = 6–9).
d, Serum total cholesterol levels (n = 3–5).
e, Serum total triacyl glyceride (TG) levels (n = 3–5).
f, Hepatic TG content (mg/g tissue weight) (n = 3–5).
g, RT–qPCR analysis of *Tnf, Ccl2, Il1b, Il6* and *Adgre1* mRNA abundance in eWAT (n = 4–6).
h, Serum concentrations of IL-1β, IL-2, IL-5, IL-6, MCP-1 and TNFα (n = 5–6).
In panels b–h, marker shapes and colors indicate the genotype and model group: **blue circles**, NC-fed WT mice (+/+); **green circles**, HFD-fed WT mice (+/+); **red circles**, STAM-MASH WT mice (+/+); **green squares**, STAM-MASH MFG-E8 heterozygous knockout mice (+/–); **purple triangles**, STAM-MASH MFG-E8 homozygous knockout mice (–/–).
The amounts of mRNAs were normalized to 18S rRNA. All data are means ± s.e.m. $^{*}P < 0.05$, $^{**}P < 0.01$, $^{***}P < 0.001$ (Student's t test or one-way ANOVA with Tukey's post hoc test).

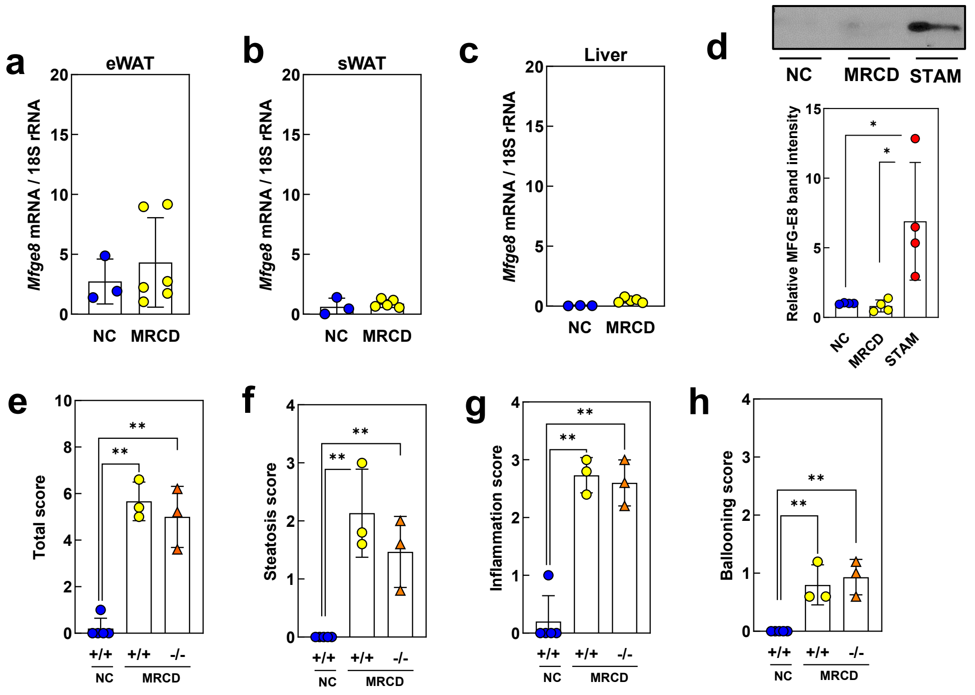

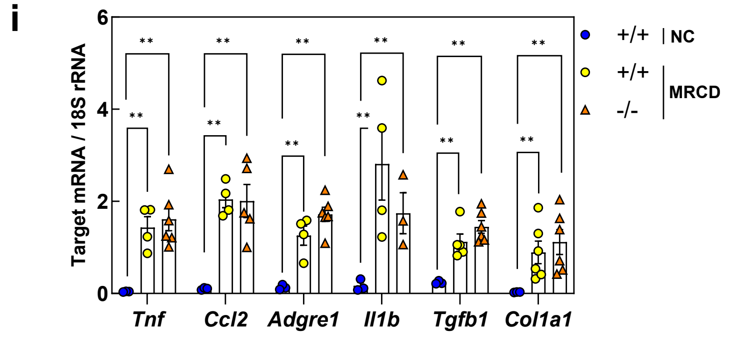

j

Analyte	WT	KO	P value
AST (IU/l)	285.5±41.9	356.5±81.5	0.46
ALT (IU/l)	346.0±45.6	279.0±22.1	0.22
TG (mg/dl)	48.0±7.9	30.7±7.6	0.15
T-Cho (mg/dl)	39.5±9.6	36.3±9.7	0.82

Supplementary Figure 4 | Phenotype of MRCD-fed MFG-E8 KO mice.

a, RT–qPCR analysis of *Mfge8* expression in eWAT (n = 3–5).
b, RT–qPCR analysis of *Mfge8* expression in sWAT (n = 3–5).
c, RT–qPCR analysis of *Mfge8* expression in liver (n = 3–5).
d, Representative immunoblot analysis of MFG-E8 in circulating EVs, with quantitative analysis of relative MFG-E8 band intensity (n = 4).
e, Total NAS scores (n = 3–5).
f, Steatosis scores (n = 3–5).
g, Inflammation scores (n = 3–5).
h, Ballooning scores (n = 3–5).
i, RT–qPCR analysis of *Tnf, Ccl2, Adgre1, Il1b, Tgfb1* and *Col1a1* mRNA expression in liver (n = 3–6).
j, Serum aspartate aminotransferase (AST), alanine aminotransferase (ALT), TG, and total cholesterol (T-Cho) levels (n = 6).
In panels **a–i**, marker shapes and colors indicate the genotype and model group: **blue circles**, NC-fed WT mice (+/+); **yellow circles**, methionine-restricted choline-deficient diet (MRCD)-fed WT mice (+/+); **red circles**, STAM-MASH WT mice; **orange triangles**, MRCD-fed MFG-E8 KO mice (–/–).
The amounts of mRNAs were normalized to 18S rRNA. All quantitative data are means ± s.e.m. *P < 0.05, **P < 0.01 (Student's t test or one-way ANOVA with Tukey's post hoc test).

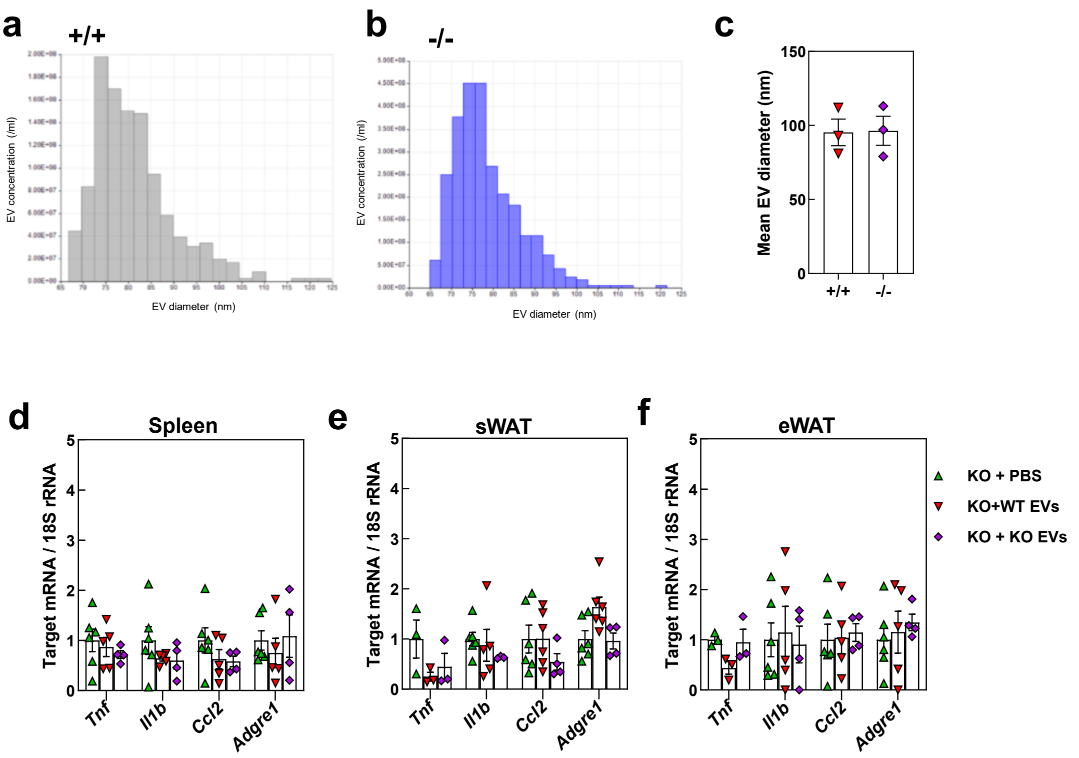

Supplementary Figure 5 | Characterization of EVs from WT or MFG-E8 KO mice and their effects in STZ-treated MFG-E8 KO mice.

a, Representative size distribution of extracellular vesicles (EVs) isolated from conditioned medium of peritoneal macrophages from WT mice (+/+).

b, Representative size distribution of EVs isolated from conditioned medium of peritoneal macrophages from MFG-E8 KO mice (–/–).

c, The mean diameter of EVs isolated from conditioned medium of peritoneal macrophages from WT (+/+) and MFG-E8 KO (–/–) mice (*n* = 3). Markers indicate the genotype of the EV source: **red inverted triangles**, WT-derived macrophages; **purple diamonds**, MFG-E8 KO derived macrophages.

d, RT–qPCR analysis of *Tnf, Il1b, Ccl2* and *Adgre1* mRNA expression in spleen (*n* = 4–6).

e, RT–qPCR analysis of *Tnf, Il1b, Ccl2* and *Adgre1* mRNA expression in sWAT (*n* = 3–6).

f, RT–qPCR analysis of *Tnf, Il1b, Ccl2* and *Adgre1* mRNA expression in eWAT (*n* = 3–6).

In panels **d–f,** marks in bar graph indicate the treatment administered to STZ-injected MFG-E8–knockout mice: **green triangles**, PBS; **red inverted triangles**, EVs derived from WT mice; **purple diamonds**, EVs derived from MFG-E8 KO mice. PBS or EVs were injected once daily for 7 days.

The amounts of mRNAs were normalized to 18S rRNA. Data in **c**–**f** are means ± s.e.m.

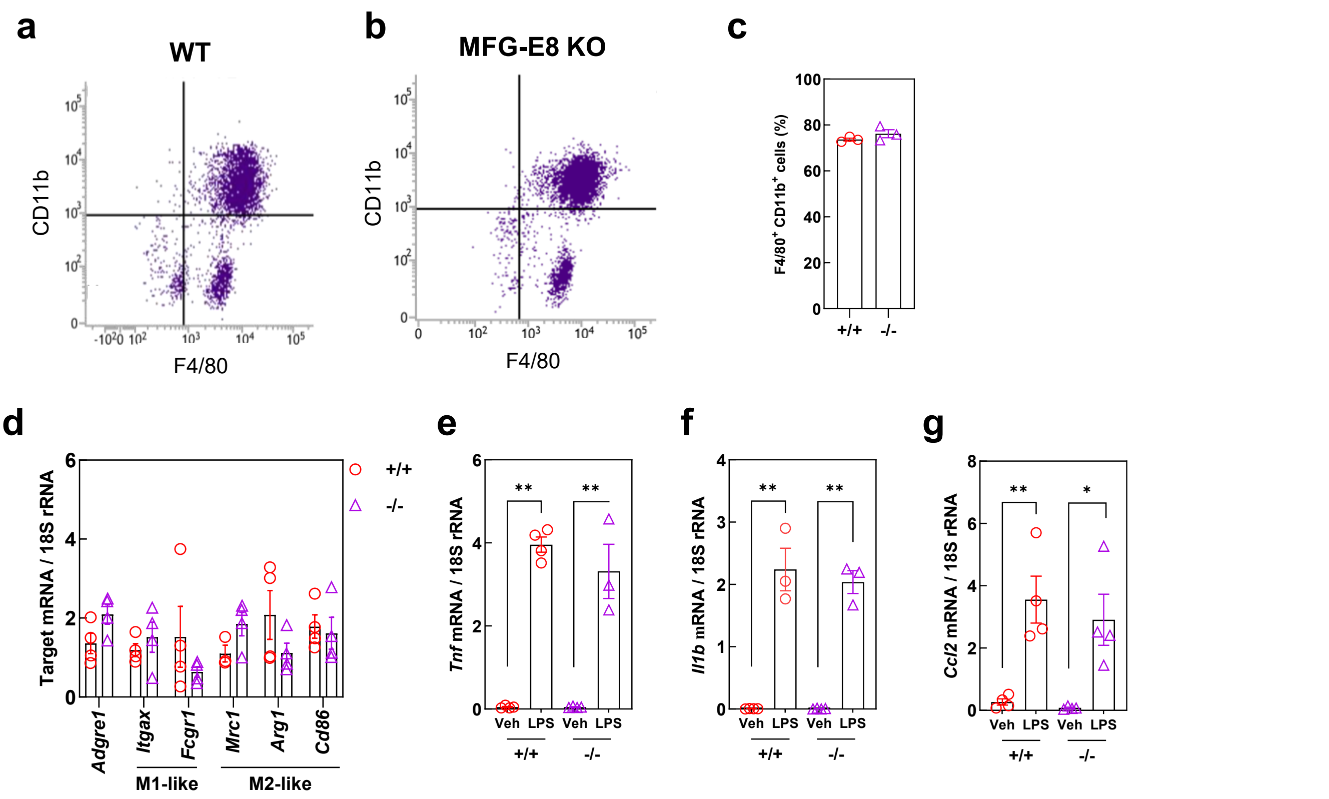

Supplementary Figure 6 | Analysis of macrophages isolated from MFG-E8 KO mice.
a, Representative flow-cytometry plot showing F4/80+CD11b+ double-positive cells in thioglycolate-elicited peritoneal cells from WT mice.
b, Representative flow-cytometry plot showing F4/80+CD11b+ double-positive cells in thioglycolate-elicited peritoneal cells from MFG-E8 KO mice.
c, Frequency of F4/80+CD11b+ double-positive cells among total peritoneal cells ($n = 3$).
d, RT–qPCR analysis of *Adgre1, Itgax, Fcgr1, Mrc1, Arg1* and *Cd86* mRNA expression in thioglycolate-elicited peritoneal cells ($n = 3–4$).
e, RT–qPCR analysis of *Tnf* mRNA expression in thioglycolate-elicited peritoneal cells treated with lipopolysaccharide (LPS; 10ng/ml for 3 hours) or PBS as a vehicle control (Veh) ($n = 3–4$).
f, RT–qPCR analysis of *Il1b* mRNA expression under the same condition as panel e ($n = 3–4$).
g, RT–qPCR analysis of *Ccl2* mRNA expression under the same condition as panel e ($n = 3–4$).
In panels c–g, marker colors indicate the genotype of peritoneal cell source: **red circles**, WT derived cells; **purple circles,** MFG-E8 KO derived cells.
The amounts of mRNAs were normalized to 18S rRNA. Data in **c** to **g** are means ± s.e.m. $*P < 0.05$, $**P < 0.01$ (Student's *t* test).

Supplementary Table. 1 | Primer sequences for RT–qPCR analysis

Gene	Forward primer sequence (5'-3')	Reverse primer sequence (5'-3')
18S rRNA	GGCCTCGAAAGAGTCCTGTA	AAACGGCTACCACATCCAAG
Mfge8	ACCCCTGTGGAGGCTCAGTA	GCAAGCCCCAGGTCTTGTAG
Tnf	TGAACTTCGGGGTGATCGGT	GTTTGCTACGACGTGGGCTAC
Ccl2	CTGTTCACAGTTGCCGGCTG	AGCTTCTTTGGGACACCTGCT
Il6	ACAACCACGGCCTTCCCTACTT	CACGATTTCCCAGAGAACATGTG
Col1a1	CATGTTCAGCTTTGTGGACCTC	GATCAAGCATACCTCGGGTTTC
Adgre1	CTTTGGCTATGGGCTTCCAGTC	GCAAGGAGGACAGAGTTTATCGTG
Il1b	GCCACCTTTTGACAGTGATGAGA	TGCCTGCCTGAAGCTCTTGT
Tgfb1	CAGACATTCGGGAAGCAGTG	CAGCCACTCAGGCGTATCAG
Mmp13	ACCTGGACAAGCAGTTCCAAA	GAAGCATGAAATGGCTTTTGC
Itgax	ACAGGACATCGCTCCCTCAC	TGAAGTGAACAGTTGGTGACACTC
Fcgr1	TAGCATCCCAGAGGCCAGTT	CCAGGGGTTCTCCTTCTGTG
Mrc1	CGGATGGCTCTGGTGTGGAA	CAGCTTGCCCTTGCCTGATG
Arg1	CAATGAAGAGCTGGCTGGTG	TGGTTGTCAGGGGAGTGTTG
Cd86	CTGTAGGCAGCACGGACTTG	CCACGGAAACAGCATCTGAG

Supplementary Table. 2 | The ARRIVE Essential 10 Check List

The ARRIVE Essential 10: Compliance Questionnaire

Use this questionnaire to evaluate how well a manuscript complies with the ARRIVE Essential 10. It can be applied to any manuscript describing comparative experiments in living animals, by assessors such as journal staff, editors, or peer reviewers.

Item		Question(s)	Answers
1	Study Design	Are all experimental and control groups clearly identified?	☒ Yes, for at least one experiment<br>☐ No
		Is the experimental unit (e.g. an animal, litter or cage of animals) clearly identified?	☒ Yes, for at least one experiment<br>☐ No
2	Sample Size	Is the exact number of experimental units in each group at the start of the study provided (e.g. in the format '*n*=')?	☒ Yes, for at least one experiment<br>☐ No
		Is the method by which the sample size was chosen explained?	☒ Yes, for at least one experiment<br>☐ No
3	Inclusion & Exclusion Criteria	Are the criteria used for including and excluding animals, experimental units, or data points provided?	☒ Yes, for at least one experiment<br>☐ No
		Are any exclusions of animals, experimental units, or data points reported, or is there a statement indicating that there were no exclusions?	☒ Yes, for at least one analysis<br>☐ No
4	Randomisation	Is the method by which experimental units were allocated to control and treatment groups described?	☒ Yes, for at least one experiment<br>☐ No
5	Blinding	Is it clear whether researchers were aware of, or blinded to, the group allocation at any stage of the experiment or data analysis?	☒ Yes, for at least one experiment<br>☐ No
6	Outcome Measures	For all experimental outcomes presented, are details provided of exactly what parameter was measured?	☒ Yes, for at least one experiment<br>☐ No
7	Statistical Methods	Is the statistical approach used to analyse each outcome detailed?	☒ Yes, for at least one analysis<br>☐ No
		Is there a description of any methods used to assess whether data met statistical assumptions?	☒ Yes, for at least one analysis<br>☐ No<br>☐ Not applicable
8	Experimental Animals	Are all species of animal used specified?	☒ Yes, for at least one experiment<br>☐ No
		Is the sex of the animals specified?	☒ Yes, for at least one experiment<br>☐ No<br>☐ Not applicable to species
		Is at least one of age, weight or developmental stage of the animals specified?	☒ Yes, for at least one experiment<br>☐ No
9	Experimental Procedures	Are both the timing and frequency with which procedures took place specified?	☒ Yes, for at least one experiment<br>☐ No
		Are details of acclimatisation periods to experimental locations provided?	☒ Yes, for at least one experiment<br>☐ No
10	Results	Are descriptive statistics for each experimental group provided, with a measure of variability (e.g. mean and SD, or median and range)?	☒ Yes, for at least one experiment<br>☐ No<br>☐ Not applicable to the type of data collected
		Is the effect size and confidence interval provided?	☐ Yes, for at least one experiment<br>☐ No<br>☒ Not applicable to the type of analysis used

Supplementary Figure 7 | Full, uncropped and unprocessed scans of all western blots used in this study.

All blots presented in the main and supplementary figures are shown here as original full scans. No cropping, rotation, contrast adjustment or lane splicing was performed. Molecular weight markers are included (hand annotated).

a

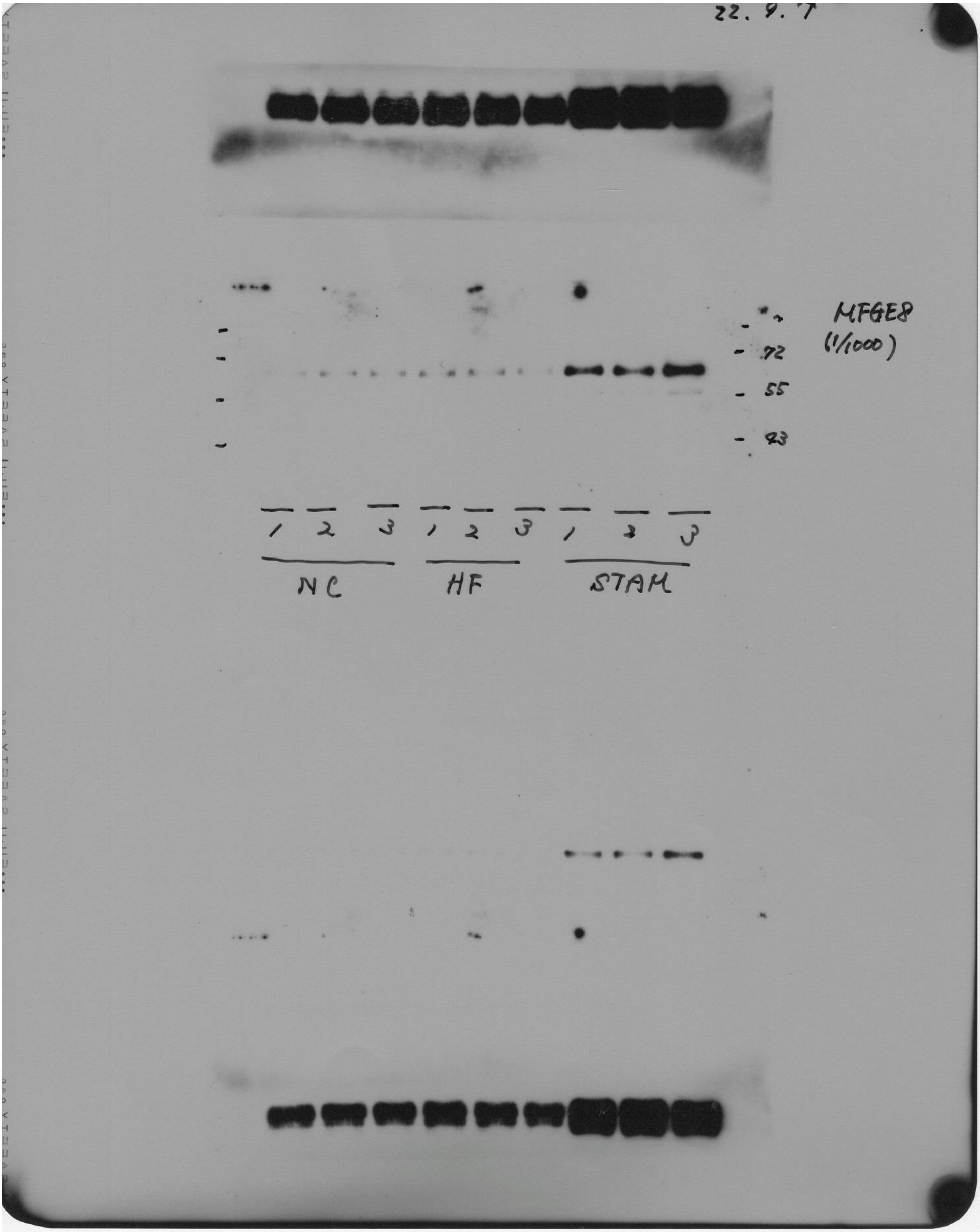

b

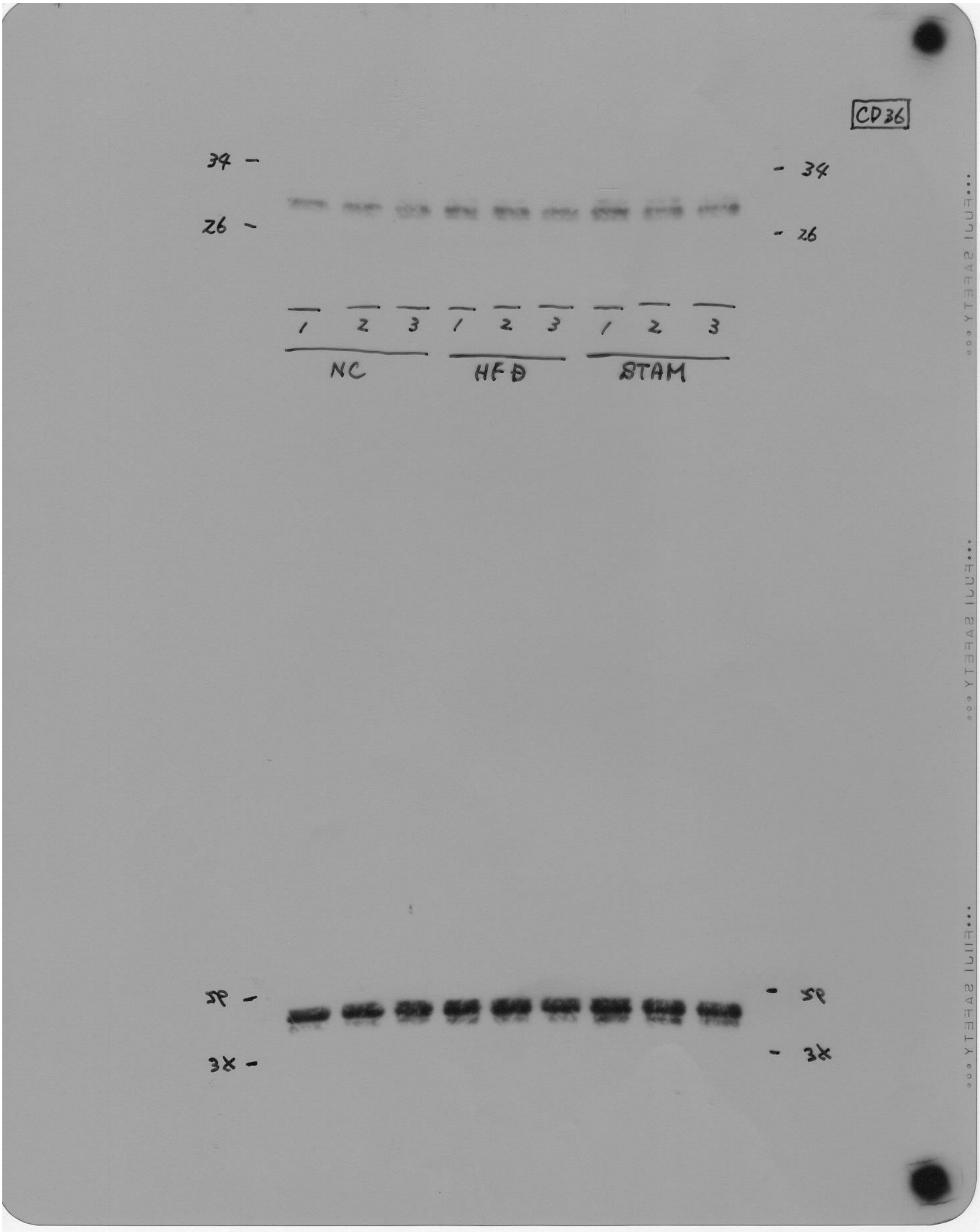

Supplementary Figure 7-1 | Uncropped blot for Fig. 1d and e.
a, MFG-E8 western blot of serum-derived EVs. This is the original full scan used to generate the cropped panel in Fig. 1d.
b, CD9 western blot of serum-derived EVs. This is the original full scan used to generate the cropped panel in Fig. 1d.
Panels a and b were used for the quantification in Fig. 1e.

c

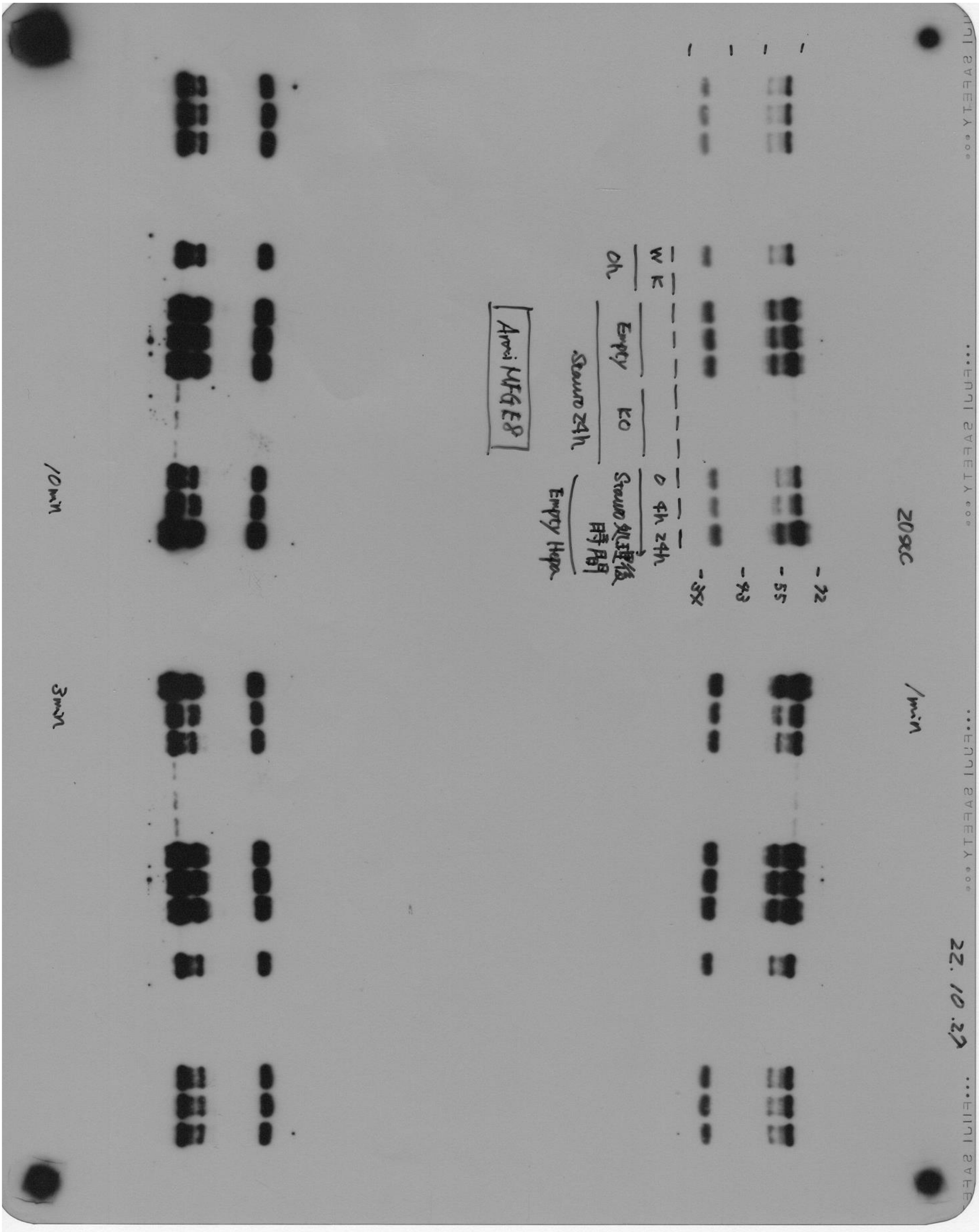

20sec
1min
3min
10min
Anti MFG E8
WK
0h
Empty
KO
Stauro 24h
0 4h 24h
Stauro処理後時間
Empty Hepa
72
55
43
34
22.10.27

d

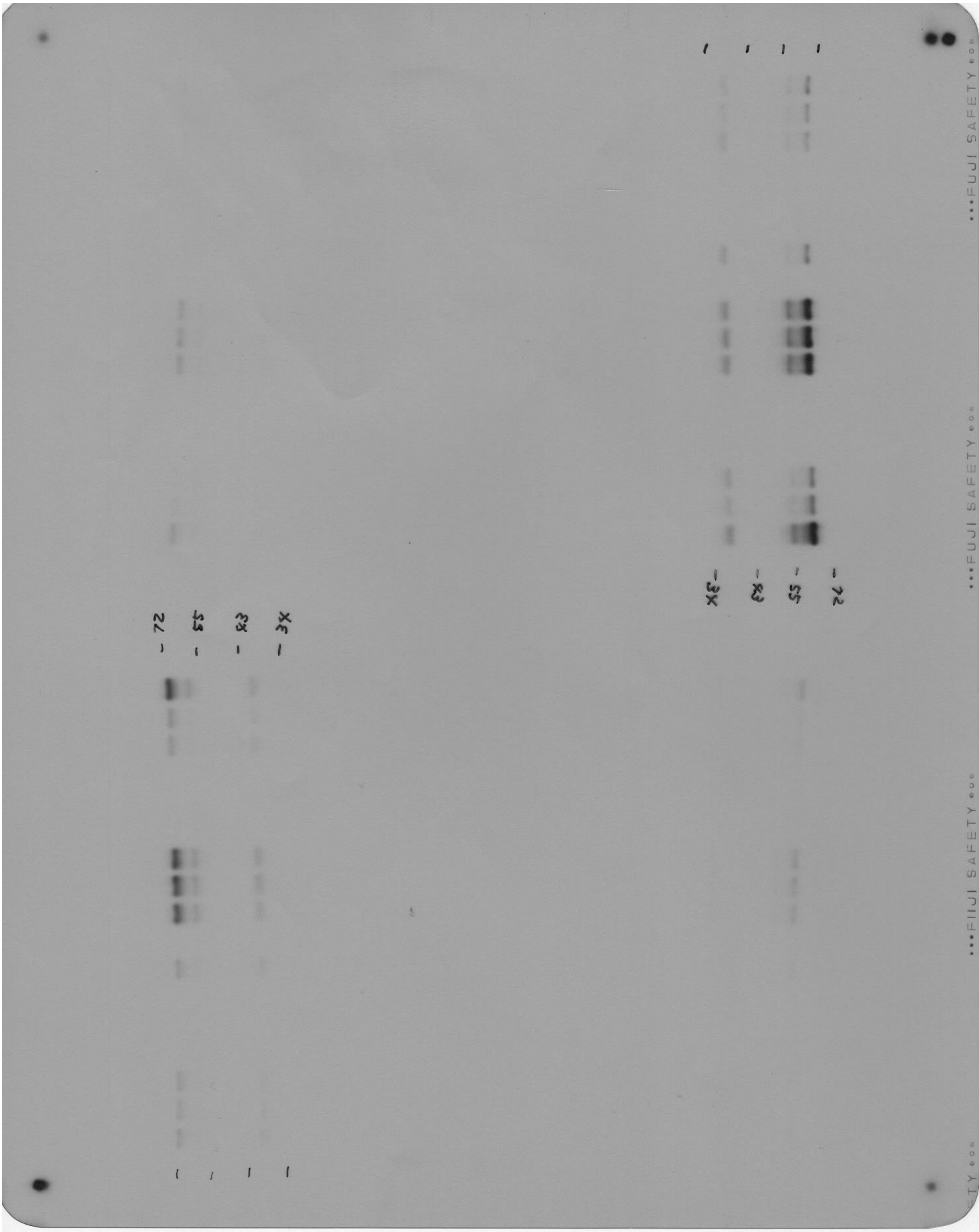

e

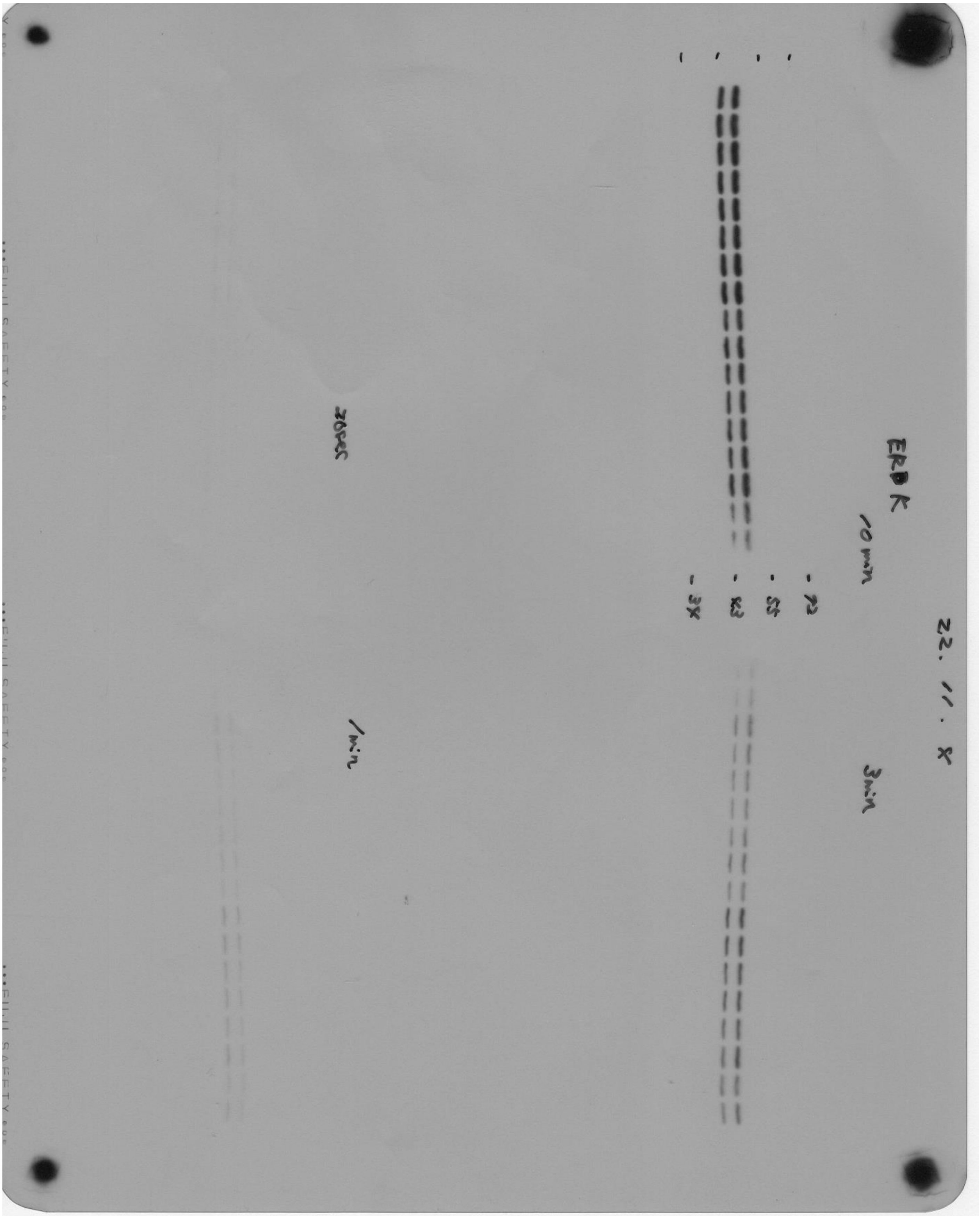

Supplementary Figure 7-2 | Uncropped blot for Fig. 4a.

c and d, MFG-E8 western blot of parental (wild types) and MFG-E8 knockout Hepa1c1c7 cells. This is the original full scan used to generate the cropped panel in Fig. 4a.

e, ERK western blot of parental (wild types) and MFG-E8 knockout Hepa1c1c7 cells. This is the original full scan used to generate the cropped panel in Fig. 4a.

.

f

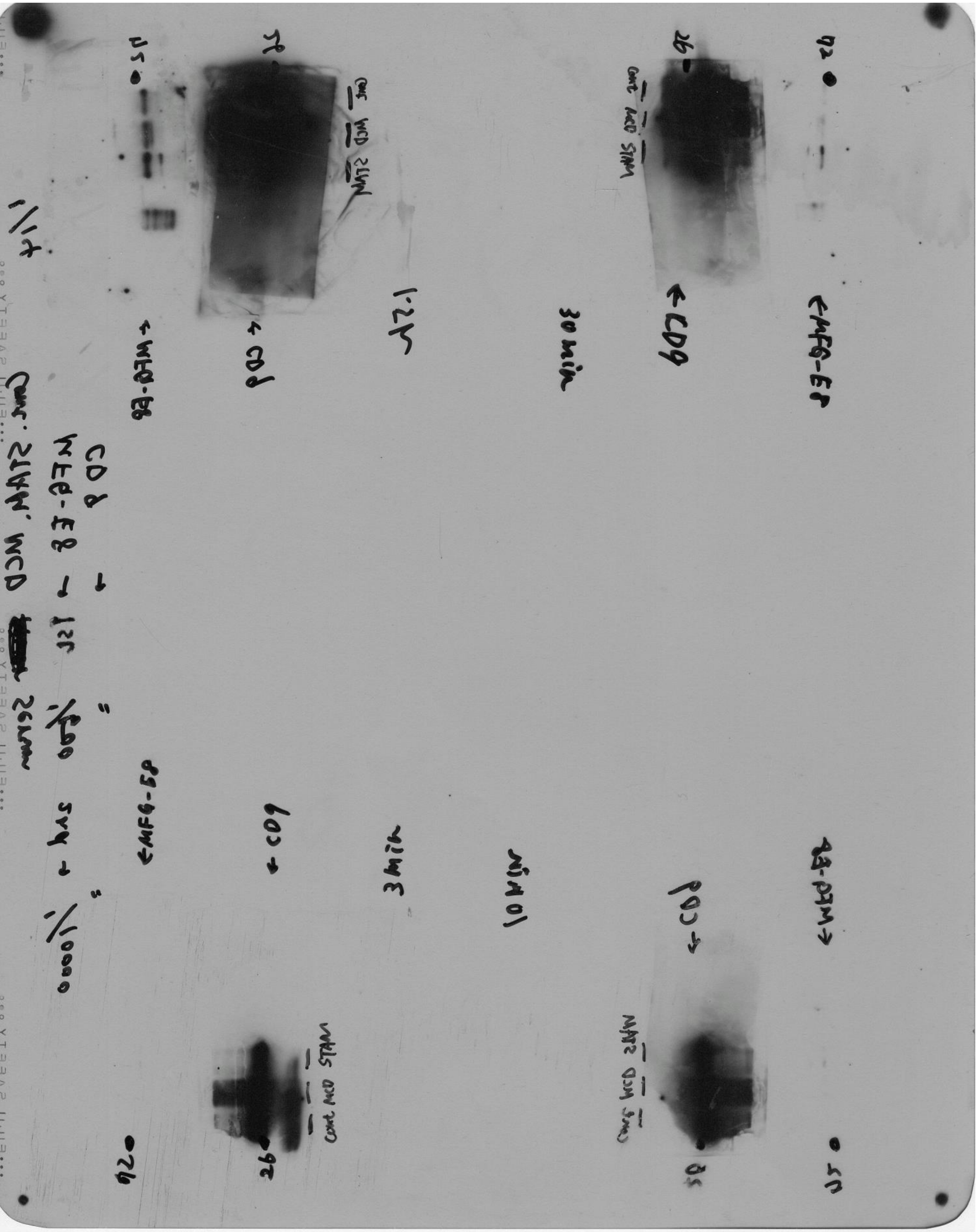

g

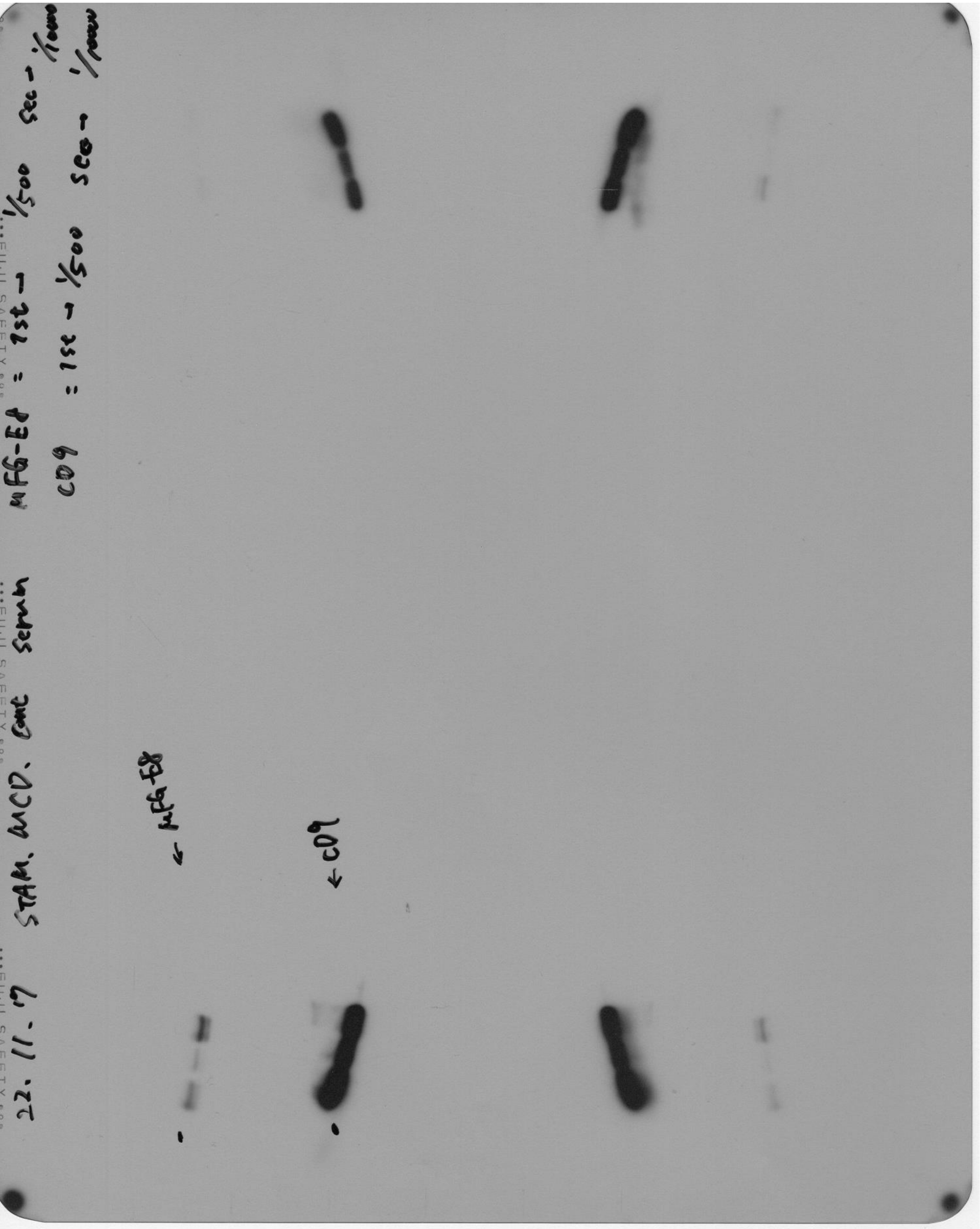

22. 11. 17 STAM. MCD. Cont Serum
MFG-E8 = 1st → 1/500 sec → 1/1000
CD9 = 1st → 1/500 sec →
← MFG-E8
← CD9

h

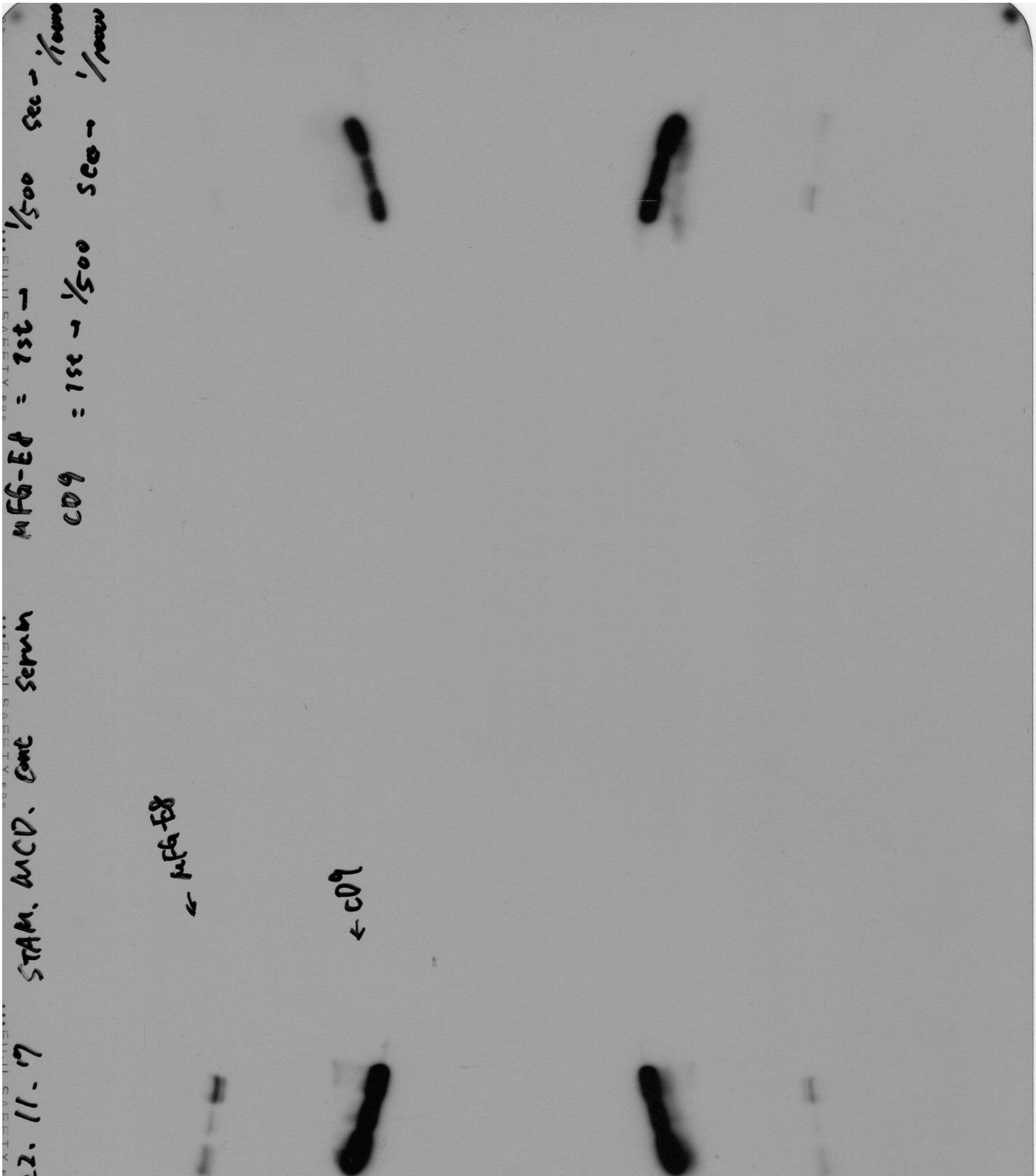
22.11.17 STAM. MCD. Cont Serum
MFG-E8 = 1st → 1/500 sec → 1/1000
CD9 = 1st → 1/500 sec → 1/1000
← MFG-E8
← CD9

i

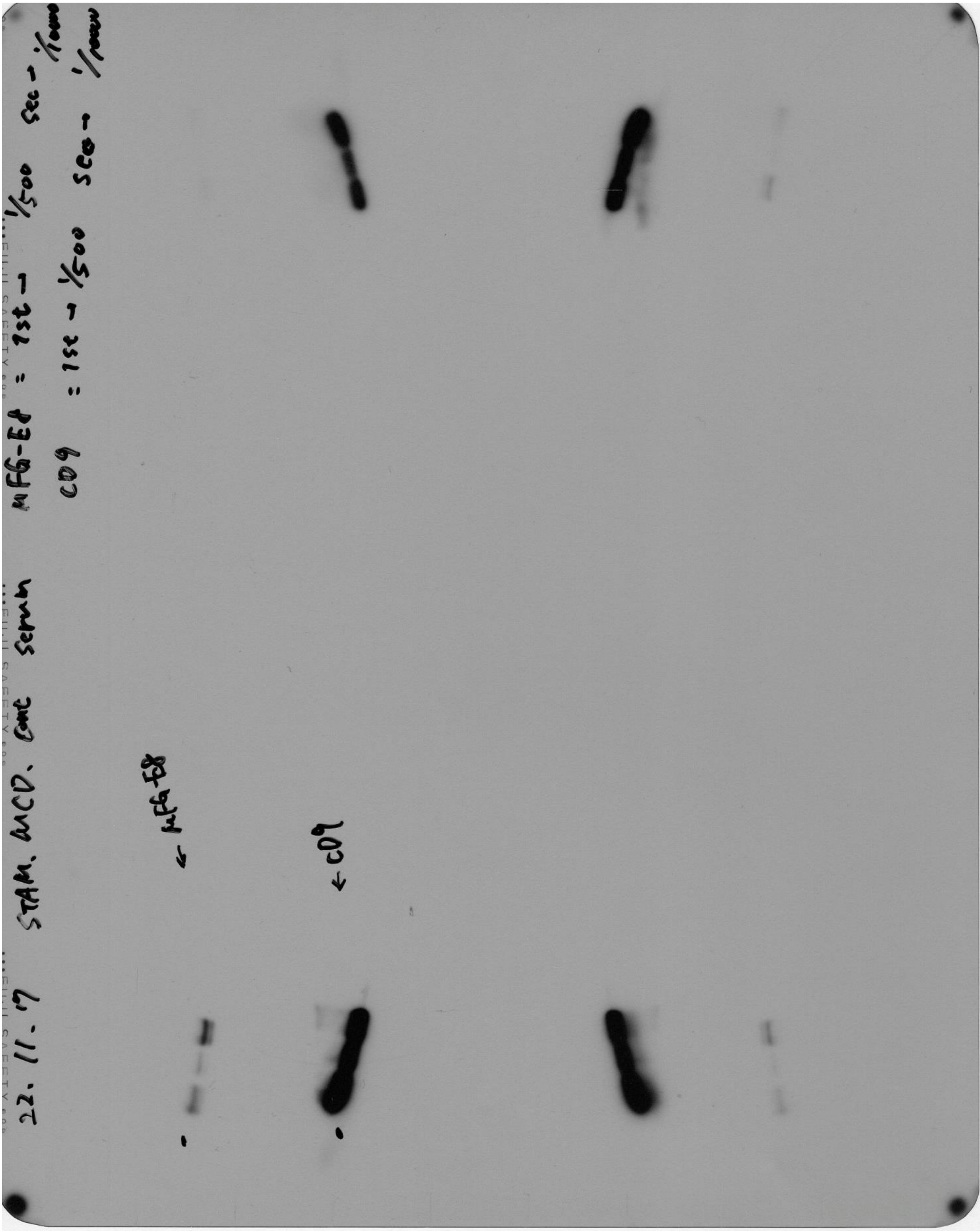
22. 11. 17 STAM. MCD. Cont Serum
MFG-E8 = 1st → 1/500 sec → 1/1000
CD9 = 1st → 1/500 sec → 1/1000
← MFG-E8
← CD9

j

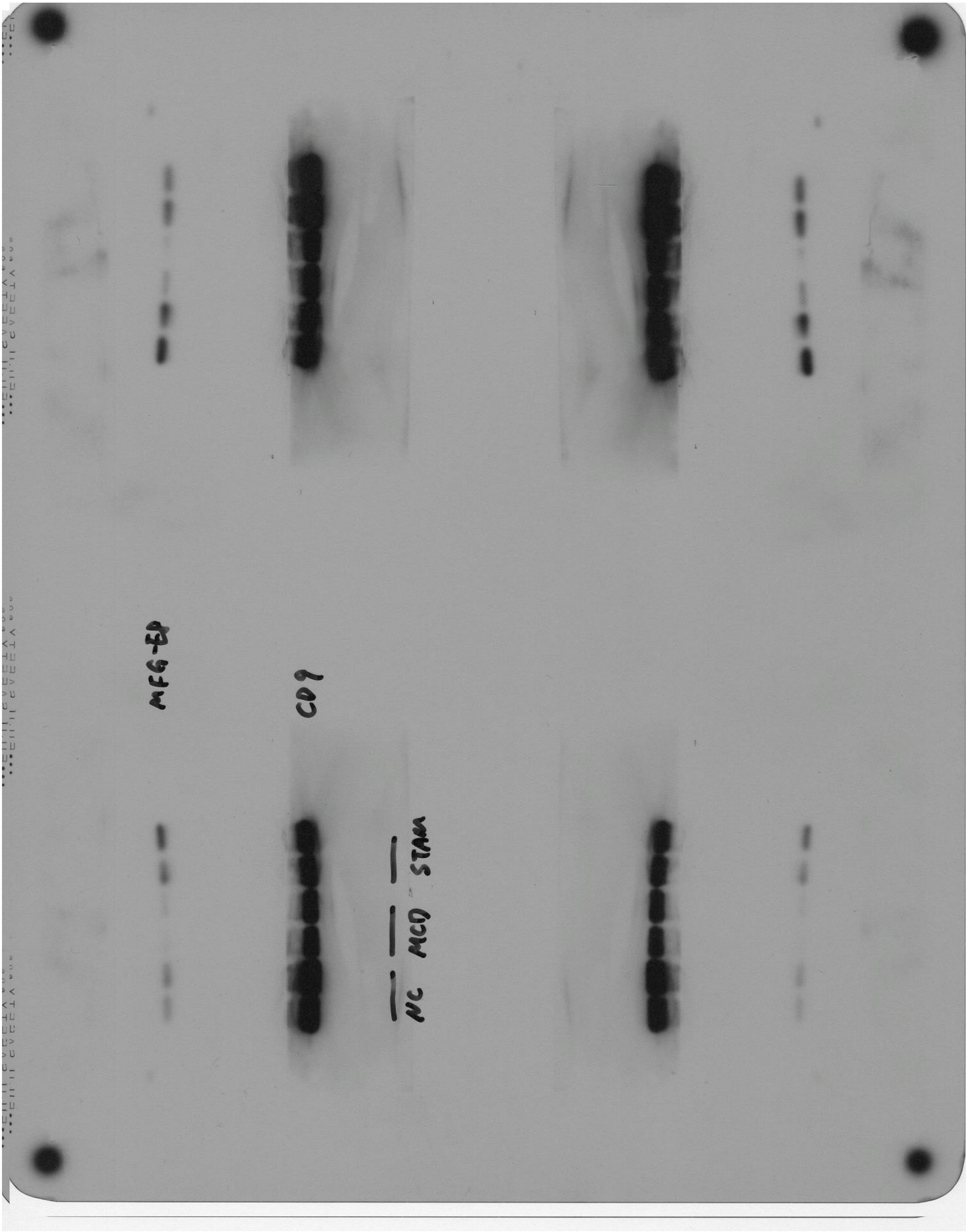

k

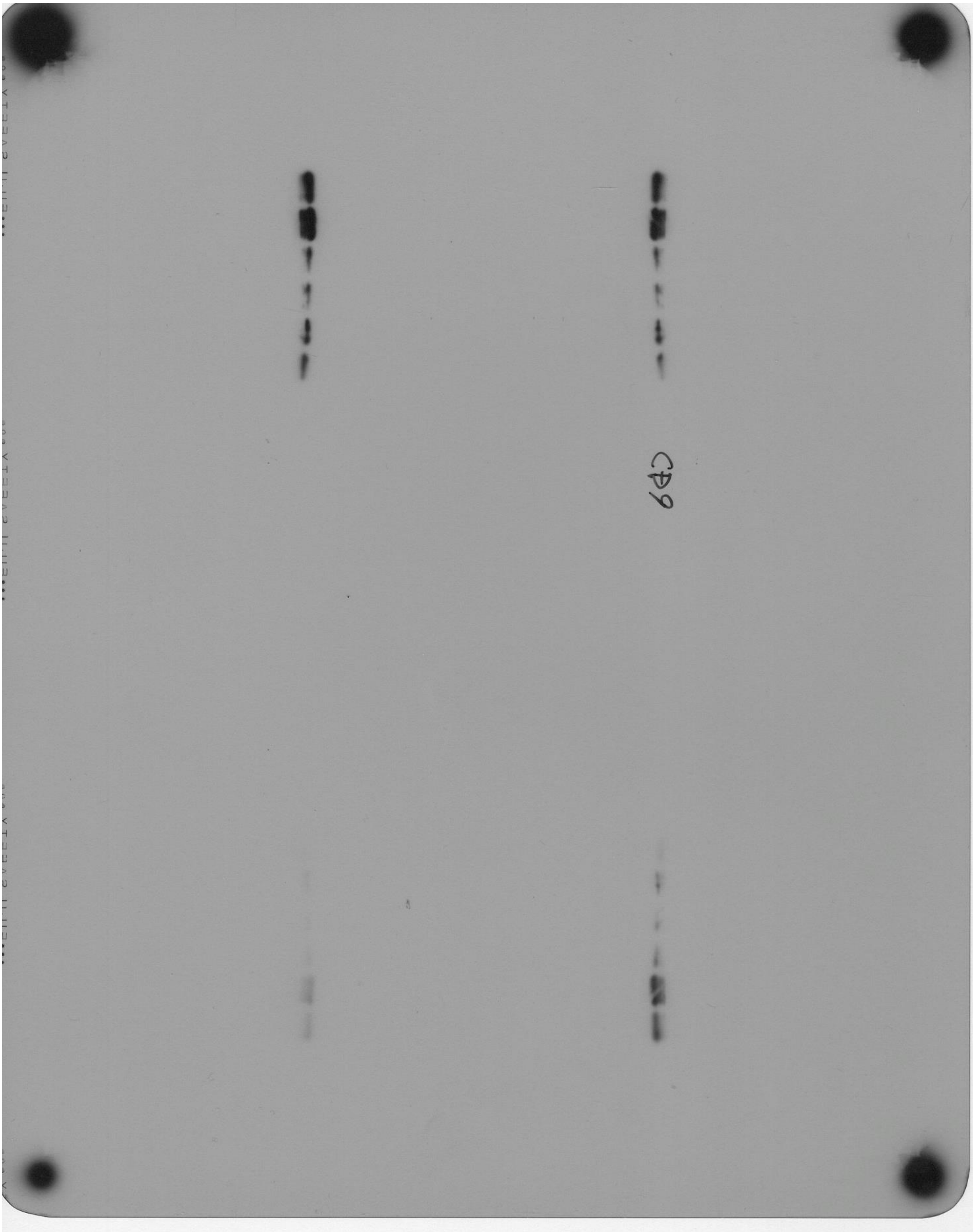

Supplementary Figure 7-3 | Uncropped blot for Supplementary Figure 4d.
f, MFG-E8 western blot of serum-derived EVs. This is the original full scan used to generate the cropped panel in Supplementary Figure 4d.
f–k, The uncropped original scans used for the quantification in Supplementary Figure 4d.
Panels f and g, h and i, and j and k represent images obtained from the same membrane but with different exposure times.
Molecular weight markers were not visible in panel h–k.
